# Pharmacological evaluation as analgesic and anti-inflammatory and molecular docking of newly synthesized nitrogen heterocyclic derivatives

**DOI:** 10.1038/s41598-025-31238-0

**Published:** 2025-12-20

**Authors:** Amina A. Abd El-Gwaad, Ahmed A. El-Rashedy, Ahmed A. Fayed, Hemat S. Khalaf

**Affiliations:** 1 Therapeutic Chemistry Department,Pharmaceutical and Drug Industries Research Institute, National Research Centre, Cairo, Egypt; 2 Natural and Microbial Products Department National Research Centre, Pharmaceutical and Drug stries Research Institute, 33 El Buhouth Street, P.O. Box 12622, Cairo, Egypt; 3 Department Organic and Medicinal Chemistry, Faculty of Pharmacy, University of Sadat City, Menoufia, 32897 Egypt; 4 Photochemistry Department, Chemical Industries Research Institute, National Research Centre, 33 El Buhouth Street, P.O. Box 12622, Cairo, Egypt

**Keywords:** Pyrazole, Pyrane, Pyrimidine, Pharmacological, Analgesic, Anti-inflammatory activities, Biochemistry, Chemical biology, Chemistry, Drug discovery

## Abstract

A new class of poly-fused pyrazolo, pyrano, and pyrimidino derivatives **2a**, **b-9a**, **b** were synthesized in this study, and their biological properties as analgesics and anti-inflammatory agents were examined. The pharmacological activity of some synthesized substances were better than those of reference controls where, compounds **6b**, **7b**, **8b** and **9b** have strong analgesic activity in comparison while, compounds **7b**, **8a** and **9b** have strong anti-inflammatory activity. Also, molecular docking, molecular dynamic (MD) simulations and thermodynamic calculation were studied. Detailed synthesis, pharmacological activity, spectroscopic analysis and molecular docking were provided.

**Supplementary Information:**

The online version contains supplementary material available at 10.1038/s41598-025-31238-0.

## Introduction

As part of our initiative to enhance the biological properties of heterocyclic compounds and facilitate their development, chalcone can be used as reactive precursors for preparing several heterocyclic compounds^1^. They are made up chemically of two aromatic rings connected by a three-carbon α,β-unsaturated carbonyl system in open-chain flavonoids. Chalcones can undergo a number of reactions to synthesize compounds with practical uses. Numerous chalcone-based substances have demonstrated Analgesic, anti-inflammatory activity through a wide range of mechanisms of action, 1, 3-diaryl-2 propen-1-ones, another name for chalcones, are a subclass of flavonoids. Chemically, two aromatic rings connected by a three-carbon α, β-unsaturated carbonyl system constitute open-chain flavonoids. Chalcones have the ability to react in a variety of ways to form useful chemicals. They are the constituents of the many flavonoids and isoflavonoids that are present in plants^2^. Chalcone is an important scaffold with unique biological and medicinal qualities^3^. Additionally, a number of heterocyclic chalcones were developed by bioisosterically substituting the aryl groups of chalcones with other heterocyclic rings, such as thiazole, thiophene, indole, chromene benzothiophene, and imidazole, which had potent analgesic and anti-inflammatory activity. For example, novel pyrimidine derivatives and heterocyclic compounds were discovered to have a variety of strong actions, including inhibitors of Tie-2 kinase^4^, HIV-1 inhibitor^5^, antimalarial^6,7^, anticancer^8^, analgesic antagonism^9^, and antiallergic activities^10^. A number of synthetic pharmacophores with antibacterial^11^, antifungal^12^, and antimycotic activities^13^ are based on the pyrimidyl motif. 2-pyrazoline derivatives have antimicrobial^14^, anti-inflammatory^15^, and antihypertensive^16^. As an extension of our ongoing studies. Introducing a pyrazolidinone ring (the azetidine and thiadiazine derivatives of isatin) were also found to possess biologically active on echinococcus multilocularis metacestodes^17^, Introducing a pyrazolidinone ring^18^ increases activity when used in penicillins and cephalosporins in place of the β-lactam ring^19^. Additionally, the second nitrogen in the five-membered ring affects the pharmacokinetic or bactericidal qualities^20^. Since their pharmacological components have been found, pyran derivatives have garnered a lot of attention. These components mostly consist of coumarins, including osthole, imperatorin, bergapten, isopimpinellin, xanthotoxol, xanthotoxin, cnidimonal, cnidimarin, and glucosides. CMC is well known for a variety of medicinal qualities, including antibacterial action^21^. Inhibition of influenza, virus sialidases^22^, mutagenic activity^23^, activity as antiviral^24^, and anti-proliferation agents^25^, sexpheromones^26^, as well as antitumor^27^, and anti-inflammatory agents^28^, and anti-osteoporotic effects^29^. Because of their interesting pharmacological characteristics, pyrimidine, pyrano and pyrazole compounds have recently received increased attention as biomolecules. This heterocyclic can be found in a number of well-known medications from different sections with a range of therapeutic activity^30,31^. From the above facts and our interest for developing new compounds possess biologically active on echinococcus multilocularis metacestodes prompted us for the synthesis of new derivatives chalcones. In addition to the synthesis of compounds, analgesic, anti-inflammatory studies in vitro and molecular docking studies are carried out.

## Results and discussion

### Chemistry

**Scheme 1:**
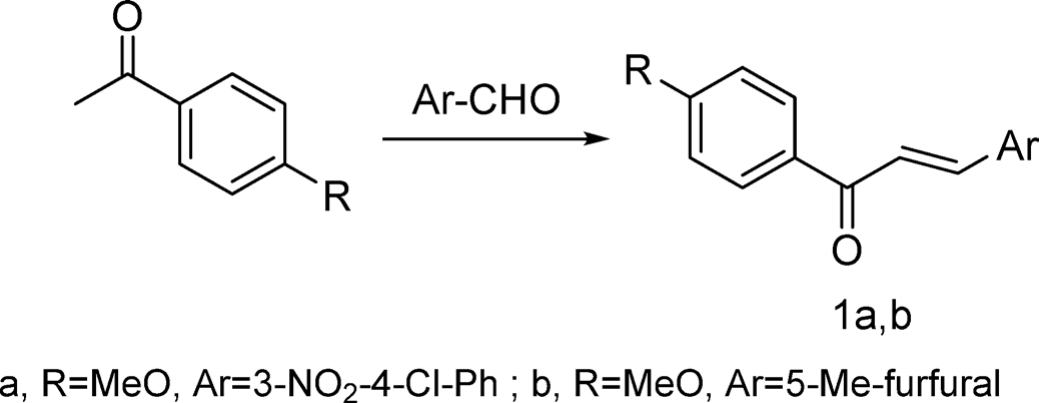
Synthesis of chalcones 1a,b

Chalcones **1a**,** b** were produced by reacting 4-methoxyacetophenone with aromatic aldehyde, namely 3-nitro-4-chlorobenzaldehyde and/or 5-methylfurfural in an alkaline medium respectively (Scheme 1).

**Scheme 2:**
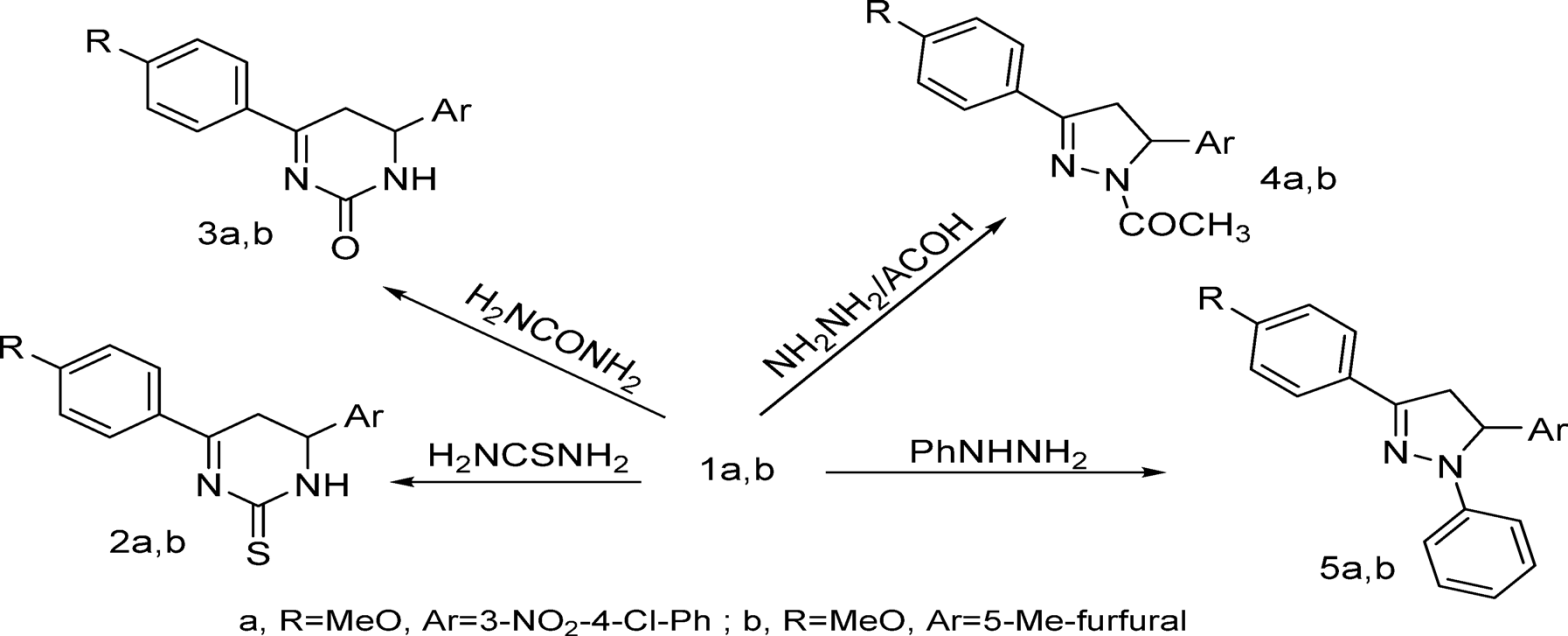
Synthesis of pyrimidinethione and pyrazolo derivatives.

Reaction of chalcones **1a**,** b** with thiourea and/or urea respectively afforded pyrimidinethione derivatives **2a**,** b** and **3a**,** b** respectively. Also, chalcone **1a**,** b** reacted with hydrazine in acetic acid and/or phenylhydrazine to afford pyrazolo derivatives **4a**,** b** and **5a**,** b** respectively (Scheme 2).

**Scheme 3:**
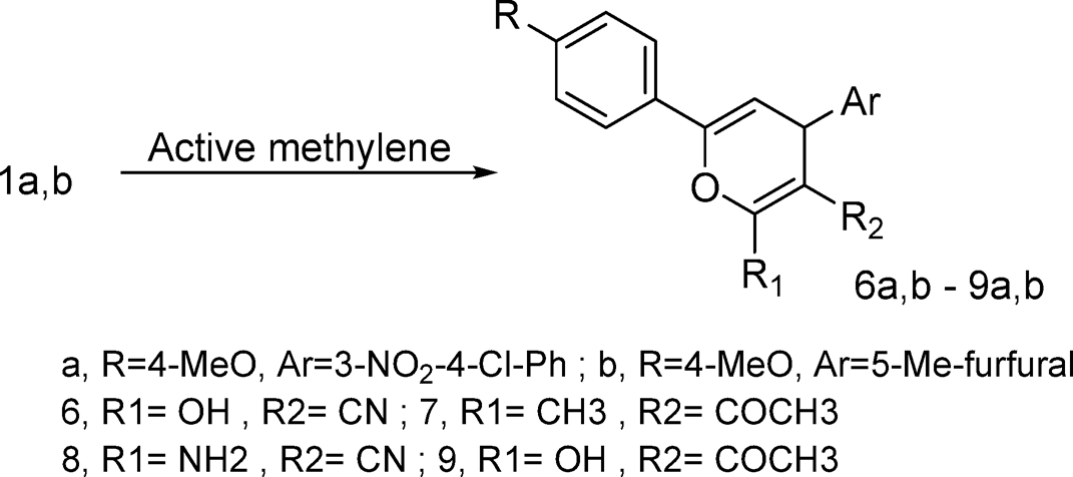
Synthesis of pyrane derivatives.

Reaction of chalcone **1a**,** b** with methylene reagents namely, ethylcyanoacetate, acetylacetone, malononitrile and/or ethylacetoacetate respectively afforded pyrane derivatives **6a**,** b − 9a**,** b** respectively (Scheme 3).

### Pharmacological activities

Two pharmacological activities were investigated, analgesic and anti-inflammatory. Compounds **2a**,** b -9a**,** b** were investigated for their analgesic and anti-inflammatory effects. These chemicals’ activity vary depending on their structure and function groups.

### Analgesic activity

Analgesic activity of tested compounds **2a**,** b-9a**,** b** after 30 min., 60 min., 90 min. and 120 min. were studied (Table 1). After 30 min. compounds **2a**,** 2b**,** 3a**,** 3b**,** 5a** and **5b** have weak analgesic activity (0.12 ± 0.01, 0.14 ± 0.02, 0.19 ± 0.01, 0.21 ± 0.03, 0.29 ± 0.03, 0.32 ± 0.02), while compounds **4a**,** 4b**,** 6a**,** 7a**,** 8a** and **9a** have intermediate activity (0.52 ± 0.01, 0.54 ± 0.02, 0.56 ± 0.02, 0.64 ± 0.02, 0.59 ± 0.02, 0.62 ± 0.03), and compounds **6b**,** 7b**,** 8b** and **9b** have strong analgesic activity (0.91 ± 0.01, 0.84 ± 0.01, 0.86 ± 0.02, 0.82 ± 0.01) respectively in comparison to Voltarene (1.00 ± 0.01). After 60 min. compounds **2a**,** 2b**,** 3a**,** 3b**,** 5a** and **5b** have weak analgesic activity (0.13 ± 0.02, 0.16 ± 0.02, 0.20 ± 0.03, 0.23 ± 0.01, 0.31 ± 0.01, 0.35 ± 0.03), while compounds **4a**,** 4b**,** 6a**,** 7a**,** 8a** and **9a** have intermediate activity (0.53 ± 0.02, 0.55 ± 0.03, 0.57 ± 0.02, 0.66 ± 0.01, 0.62 ± 0.02, 0.63 ± 0.02), and compounds **6b**,** 7b**,** 8b** and **9b** have strong analgesic activity (0.92 ± 0.02, 0.85 ± 0.01, 0.91 ± 0.03, 0.85 ± 0.02) respectively in comparison to Voltarene (1.00 ± 0.01). After 90 min. compounds **2a**,** 2b**,** 3a**,** 3b**,** 5a** and **5b** have weak analgesic activity (0.15 ± 0.01, 0.17 ± 0.03, 0.21 ± 0.01, 0.24 ± 0.02, 0.32 ± 0.03, 0.36 ± 0.01), while compounds **4a**,** 4b**,** 6a**,** 7a**,** 8a** and **9a** have intermediate activity (0.56 ± 0.01, 0.57 ± 0.02, 0.60 ± 0.02, 0.68 ± 0.03, 0.63 ± 0.01, 0.65 ± 0.01), and compounds **6b**,** 7b**,** 8b** and **9b** have strong analgesic activity (0.94 ± 0.01, 0.87 ± 0.02, 0.92 ± 0.02, 0.89 ± 0.03) respectively in comparison to Voltarene (1.00 ± 0.01). After 120 min., compounds **2a**,** 2b**,** 3a**,** 3b**,** 5a** and **5b** have weak analgesic activity (0.18 ± 0.03, 0.19 ± 0.02, 0.23 ± 0.01, 0.26 ± 0.02, 0.34 ± 0.02, 0.39 ± 0.02), while compounds **4a**,** 4b**,** 6a**,** 7a**,** 8a** and **9a** have intermediate activity (0.58 ± 0.01, 0.61 ± 0.03, 0.62 ± 0.01, 0.71 ± 0.01, 0.69 ± 0.01, 0.68 ± 0.02), and compounds **6b**,** 7b**,** 8b** and **9b** have strong analgesic activity (0.96 ± 0.03, 0.90 ± 0.02, 0.94 ± 0.03, 0.92 ± 0.01) respectively in comparison to Voltarene (1.00 ± 0.01) (Table 1).

**Table 1:** Analgesic activities of newly synthesized compounds.

Compound No.	Analgesic activities of some newly synthesized compounds after			
	30 min.	60 min.	90 min.	120 min.
**2a**	0.12 ± 0.01	0.13 ± 0.02	0.15 ± 0.01	0.18 ± 0.03
**2b**	0.14 ± 0.02	0.16 ± 0.02	0.17 ± 0.03	0.19 ± 0.02
**3a**	0.19 ± 0.01	0.20 ± 0.03	0.21 ± 0.01	0.23 ± 0.01
**3b**	0.21 ± 0.03	0.23 ± 0.01	0.24 ± 0.02	0.26 ± 0.02
**4a**	0.52 ± 0.01	0.53 ± 0.02	0.56 ± 0.01	0.58 ± 0.01
**4b**	0.54 ± 0.02	0.55 ± 0.03	0.57 ± 0.02	0.61 ± 0.03
**5a**	0.29 ± 0.03	0.31 ± 0.01	0.32 ± 0.03	0.34 ± 0.02
**5b**	0.32 ± 0.02	0.35 ± 0.03	0.36 ± 0.01	0.39 ± 0.02
**6a**	0.56 ± 0.02	0.57 ± 0.02	0.60 ± 0.02	0.62 ± 0.01
**6b**	0.91 ± 0.01	0.92 ± 0.02	0.94 ± 0.01	0.96 ± 0.03
**7a**	0.64 ± 0.02	0.66 ± 0.01	0.68 ± 0.03	0.71 ± 0.01
**7b**	0.84 ± 0.01	0.85 ± 0.01	0.87 ± 0.02	0.90 ± 0.02
**8a**	0.59 ± 0.02	0.62 ± 0.02	0.63 ± 0.01	0.69 ± 0.01
**8b**	0.86 ± 0.02	0.91 ± 0.03	0.92 ± 0.02	0.94 ± 0.03
**9a**	0.62 ± 0.03	0.63 ± 0.02	0.65 ± 0.01	0.68 ± 0.02
**9b**	0.82 ± 0.01	0.85 ± 0.02	0.89 ± 0.03	0.92 ± 0.01
**Voltarene**	1.00 ± 0.01	1.00 ± 0.01	1.00 ± 0.01	1.00 ± 0.01

### Anti-inflammatory activity

Tested compounds **2a**,** b-9a**,** b** were examined for their anti-inflammatory properties after 3 h. and 6 h. (Table 2) after 3 h. compounds **2a**,** 2b**,** 3a** and **5b** have weak anti-inflammatory activity (23 ± 0.11, 25 ± 0.13, 32 ± 0.12 and 21 ± 0.13), while compounds **3b**,** 4a**,** 4b**,** 5a**,** 6a**,** 6b**,** 7a**,** 8a** and **9a** have intermediate activity (53 ± 0.13, 51 ± 0.11, 55 ± 0.11, 52 ± 0.12, 57 ± 0.13, 61 ± 0.12, 62 ± 0.12, 65 ± 0.11 and 69 ± 0.12), and compounds **7b**,** 8a** and **9b** have strong anti-inflammatory (82 ± 0.13, 85 ± 0.11 and 86 ± 0.12) respectively in comparison to flurbiprofen (100 ± 0.13).

After 6 h. compounds **2a**,** 2b**,** 3a** and **5b** have weak anti-inflammatory activity (24 ± 0.13, 27 ± 0.12, 33 ± 0.13 and 25 ± 0.11), while compounds **3b**,** 4a**,** 4b**,** 5a**,** 6a**,** 6b**,** 7a**,** 8a** and **9a** have intermediate activity (54 ± 0.12, 53 ± 0.13, 57 ± 0.11, 54 ± 0.12, 58 ± 0.13, 62 ± 0.13, 64 ± 0.11, 68 ± 0.13 and 72 ± 0.13), and compounds **7b**,** 8a** and **9b** have strong anti-inflammatory activity (84 ± 0.12, 87 ± 0.12 and 89 ± 0.11) respectively in comparison to flurbiprofen (100 ± 0.12). (Table 2).

**Table 2:** Anti-inflammatory activities of some newly synthesized compounds.

Compound No.	Post treatment 3 h. %	Post treatment 6 h. %
**2a**	23 ± 0.11	24 ± 0.13
**2b**	25 ± 0.13	27 ± 0.12
**3a**	32 ± 0.12	33 ± 0.13
**3b**	53 ± 0.13	54 ± 0.12
**4a**	51 ± 0.11	53 ± 0.13
**4b**	55 ± 0.11	57 ± 0.11
**5a**	52 ± 0.12	54 ± 0.12
**5b**	21 ± 0.13	25 ± 0.11
**6a**	57 ± 0.13	58 ± 0.13
**6b**	61 ± 0.12	62 ± 0.13
**7a**	62 ± 0.12	72 ± 0.13
**7b**	82 ± 0.13	84 ± 0.12
**8a**	65 ± 0.11	68 ± 0.13
**8b**	85 ± 0.11	87 ± 0.12
**9a**	69 ± 0.12	72 ± 0.13
**9b**	86 ± 0.12	89 ± 0.11
**Flurbiprofen**	100 ± 0.13	100 ± 0.12

### Molecular docking

The molecular docking studies against the COX-2 enzyme (PDB: 6COX) reveal a compelling correlation with the experimental biological activities, providing a plausible mechanistic basis for the observed effects. The docking scores demonstrate that the most potent compounds—6b, 8b, 9b, and 7b—which exhibited strong analgesic and anti-inflammatory activity in vivo, also possess the most favorable (most negative) binding energies of -14.27, -13.25, -13.82, and − 12.23 kcal/mol, respectively. Notably, all these “b” series compounds bind more strongly to the COX-2 active site than the reference drug Flurbiprofen (-11.90 kcal/mol). Conversely, compounds with weaker biological activity, such as 5a (weak analgesic/anti-inflammatory), correspondingly showed one of the least favorable docking scores (-11.42 kcal/mol). This strong positive correlation between high docking affinity and enhanced pharmacological potency strongly suggests that the primary mechanism of action for these novel compounds is through effective inhibition of the COX-2 enzyme, thereby explaining their significant anti-inflammatory and analgesic effects. (Table 3, S1)

**Table 3:** Molecular Docking results and binding free energies of synthesized compounds and reference drugs against COX-2 (PDB: 6COX).

Compound No.	Docking Score (kcal/mol)
**2a**	-12.87
**2b**	-12.41
**3a**	-12.60
**3b**	-12.90
**4a**	-12.06
**4b**	-12.78
**5a**	-11.42
**5b**	-12.67
**6a**	-12.08
**6b**	-14.27
**7a**	-11.82
**7b**	-12.23
**8a**	-12.84
**8b**	-13.25
**9a**	-12.30
**9b**	-13.82
**Flurbiprofen**	-11.90
**Diclofenac**	-12.32

### Molecular dynamic and system stability

**Fig. 1:**
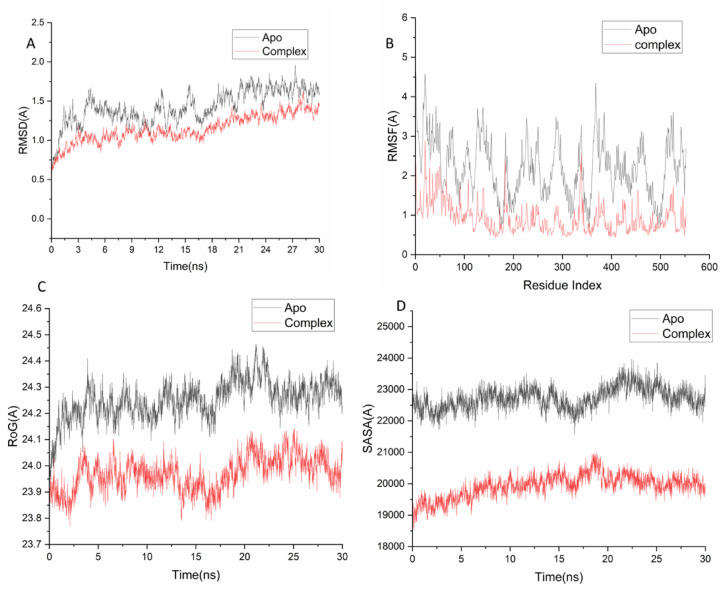
[A] RMSD of Cα atoms of the protein backbone atoms. [B] RMSF of each residue of the protein backbone Cα atoms of protein residues (c) ROG of Cα atoms of protein residues; (d) solvent accessible surface area (SASA) of the Cα of the backbone atoms relative (black) to the starting minimized over 30 ns for the catalytic binding site with 6b – Cox-2 complex system (red).

Molecular dynamics (MD) simulations were conducted to evaluate the binding performance, molecular interactions, and stability of the extracted compounds within the protein’s active site^32^. Validating system stability is crucial for identifying aberrant motions and potential simulation artifacts. To this end, the Root-Mean-Square Deviation (RMSD) was analyzed, revealing average values of 1.46 ± 0.22 Å for the apo-protein and 1.15 ± 0.17 Å for the 6b-COX-2 complex (Fig. 1A). Protein flexibility upon ligand binding was assessed via Root-Mean-Square Fluctuation (RMSF) to examine residue behavior and ligand interactions^33^. The average RMSF values were 2.21 ± 0.71 Å for the apo-protein and 0.92 ± 0.38 Å for the 6b complex (Fig. 1B), indicating reduced residue fluctuations in the ligand-bound state. The Radius of Gyration (Rg) was calculated to determine overall system compactness and stability^34^. The average Rg values were 24.25 ± 0.07 Å (apo) and 23.96 ± 0.06 Å (6b complex) (Fig. 1A), suggesting a more rigid structure for the ligand-bound system.Finally, the Solvent Accessible Surface Area (SASA) was measured to probe the compactness of the hydrophobic core, a key factor in biomolecular stability^35^. The average SASA values were 22717.44 Å² for the apo-protein and 11925.65 Å² for the 6b complex. The combined data from RMSD, RMSF, Rg, and SASA analyses confirm that the 6b complex remains stable and intact within the catalytic binding site.

### Binding interaction mechanism based on binding free energy calculation

The molecular mechanics generalized Born surface area (MM/GBSA) method is a widely used approach for estimating binding free energies, often providing more reliable predictions than docking scores alone by integrating continuum solvation models^36^. Utilizing the MM-GBSA implementation in AMBER18, the binding free energy for the complex was calculated by analyzing snapshots extracted from the molecular dynamics trajectories. The results, presented in Table 4, demonstrate that all computed energy components, with the exception of the solvation free energy (ΔGsolv), exhibited strongly negative values, which is indicative of a favorable binding interaction.

**Table 4:** Shows the calculated energy binding for the compound against the catalytic binding site of COX-2 target receptor. ∆EvdW = van der Waals energy; ∆Eele = electrostatic energy; ∆Gsolv = solvation free energy; ∆Gbind = calculated total binding free energy.

Energy Components (kcal/mol)					
Human cyclooxygenase-2 (COX-2)					
Complex	ΔE_vdW_	ΔE_elec_	ΔG_gas_	ΔG_solv_	ΔG_bind_
**6b - COX-2**	-45.06 ± 0.69	-38.41 ± 0.22	-83.47 ± 0.11	39.57 ± 0.79	-43.89 ± 0.73

A detailed energy decomposition analysis revealed that the binding of the 6b compound to the target protein is primarily driven by a highly favorable van der Waals energy component. (Table 3)

### Identification of the critical residues responsible for ligands binding

**Fig. 2:**
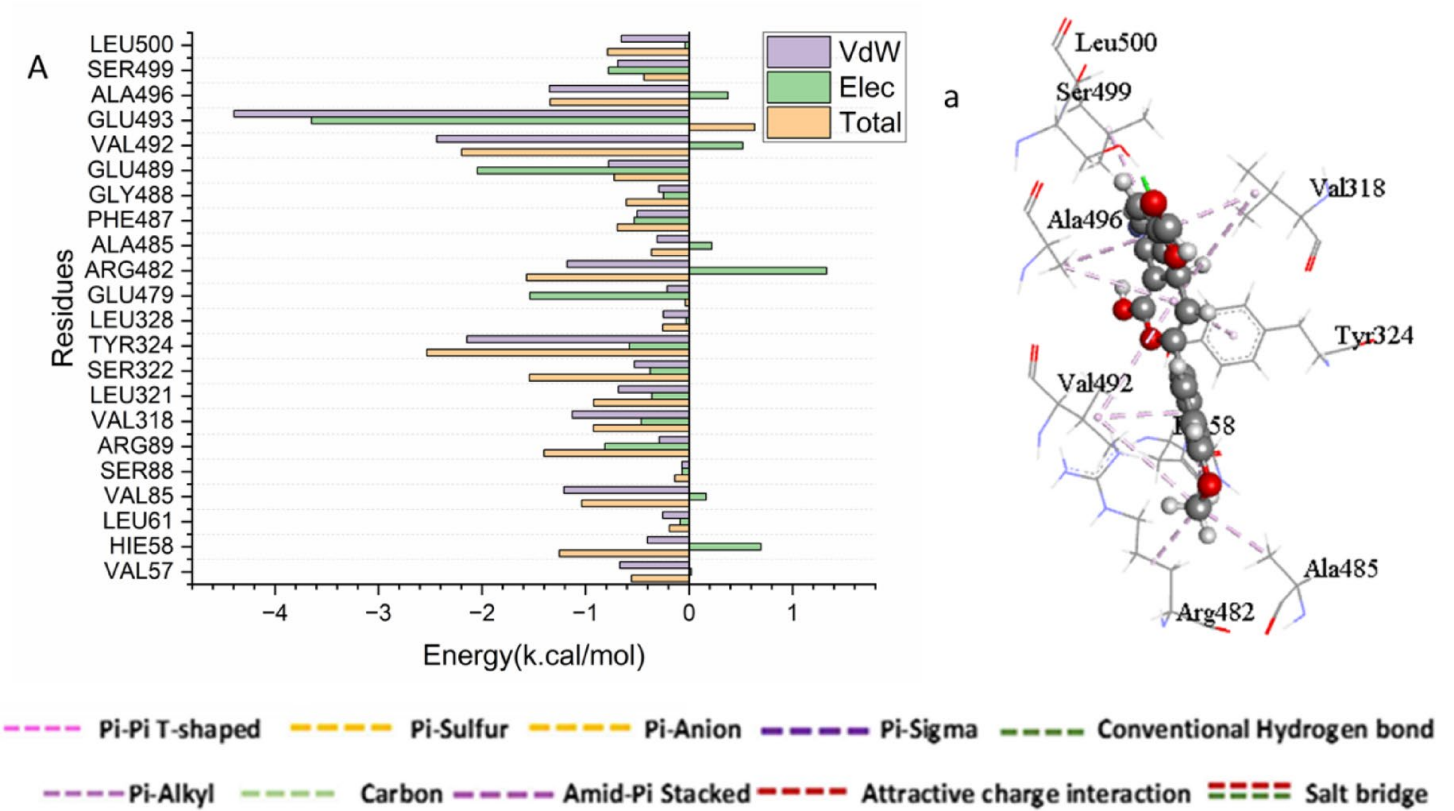
Per-residue decomposition plots showing the energy contributions to the binding and stabilization of 6b into catalytic binding site of Human cyclooxygenase-2 (COX-2) [A], Corresponding inter-molecular interactions are shown [a].

To identify key residues involved in binding, the total binding energy was decomposed into per-residue contributions. As shown in Fig. 2, the favorable binding of the 6b compound to the cyclooxygenase-2 (COX-2) catalytic site is predominantly driven by interactions with the following residues Val 57 (-0.669 kcal/mol), Hie 58 (-0.403 kcal/mol), Leu 61 (-0.253 kcal/mol), Val 85 ( -1.209 kcal/mol), Arg 89 (-0.287 kcal/mol),, Val 318 (-1.128 kcal/mol), Leu 321(-0.683 kcal/mol), Ser 322 (-0.529 kcal/mol), Tyr 324 (-2.145 kcal/mol), Leu 328 (-0.249 kcal/mol), Glu 479 (-0.212 kcal/mol), Arg 482 (-1.178 kcal/mol), Phe 487(-0.502 kcal/mol), Gly 488 (-0.292 kcal/mol), Glu 489 (-0.777 kcal/mol), Glu 493 (-4.396 kcal/mol),, Ala 496 (-1.348 kcal/mol), and Leu 500 (-0.655 kcal/mol).

## Materials and methods

All melting points were decided in open glass capillaries using an Electro thermal IA 9000 Series digital melting point apparatus (Electro thermal, Essex, UK) and are uncorrected. Elemental analyses were performed with all final compounds with an Elementary, Vario EL, Micro analytical Unit, Cairo University, Cairo Egypt, and were in good agreement (0.2%) with the calculated values. IR (KBr) spectra were recorded on a Shimadzu 408 spectrophotometer as a solid suspended in a potassium bromide disk. ^1^H and^13^C NMR spectra were recorded on a Varian 500 MHZ”. “All chemical shifts were reported as d (ppm) scale using TMS as the standard, and coupling-constant values were given in Hz. The mass spectra (EI) were run at 70 eV with a Finnegan SSQ 7000 spectrometer (Thermo Instrument System Incorporated, USA), m/z values are indicated in Dalton.

### Synthesis and characterization

#### Synthesis of compounds 1a, b

To a solution of 4-methoxyacetophenone (0.01 mol) in ethanol (30 mL) and potassium carbonate (0.52 g) was added over a period of 30 min. Then the corresponding aldehyde (0.01 mol) was slowly added to the reaction mixture with stirring at room temperature for 12 h. Then the reaction mixture was evaporated under vacuum, The solid that formed was filtered, dried, and recrystallized from proper solvent to afford compounds 1a and 1b respectively.

#### (E)-3-(4-chloro-3-nitrophenyl)-1-(4-methoxyphenyl)prop-2-en-1-one (1a)

From ethanol (90%); mp 172–174 °C; IR ν cm^− 1^: 1680 (C = C); 1710 (C = O ketone); ^1^H-NMR (DMSO-d6, δ, ppm): 3.28 (s, 3 H, OCH_3_), 6.93–7.58 (, 11 H aromatic), 7.91 (d, 1H, CH = CH), 8.21 (d, 1H, CH = CH); ^13^C-NMR (DMSO-d6, δ, ppm): 54.62, 115.32, 118.17, 121.21, 123.31, 126.35, 127.54, 129.31, 130.38, 133.23, 134.52, 136.26, 138.47, 141.36, 143.62, 144.38, 147.21, 148.26, 150.36, 153.16, 156.34, 164.28 (22 C); MS: m/z (%): 393 (M^+^, 32) and 208 (100, base peak); Anal. calcd for C_22_H_16_ClNO_4_ (393.82): C, 67.10; H, 9.00; N, 3.56; Found: C, 66.92; H, 3.89; N, 3.37.

#### (E)-1-(4-methoxyphenyl)-3-(5-methylfuran-2-yl)prop-2-en-1-one (1b)

From methanol (86%); mp 183–185 °C; IR ν cm^− 1^: 1640 (C = C); 1715 (C = O ketone); ^1^H-NMR (DMSO-d6, δ, ppm): 2.74 (s, 3 H, CH_3_), 3.16 (s, 3 H, OCH_3_), 7.13–7.69 (m, 10 H, 8Haromatic, 2 H furane), 7.82 (d, 1H, CH = CH), 8.14 (d, 1H, CH = CH); ^13^C-NMR (DMSO-d6, δ, ppm): 36.51, 53.36, 116.31, 119.13, 121.26, 124.33, 128.29, 129.37, 131.81, 133.34, 134.25, 136.19, 138.28, 140.32, 144.61, 146.24, 148.22, 152.32, 154.31, 158.27, 167.14 (21 C); MS: m/z (%): 318 (M^+^, 41) and 213 (100, base peak); Anal. calcd for C_21_H_18_O_3_ (318.43): C, 79.23; H, 5.70; O, 15.08; Found: C, 79.02; H, 5.49; O, 14.89.

#### Synthesis of compounds 2a, b and 3a, b

A mixture of the chalcone 1a and /or 1b respectively (0.01 mol), thiourea and /or urea (0.01 mol), and potassium carbonate (0.08 mol) in ethanol (30 mL) was refluxed for 6 h. The reaction mixture was concentrated, cooled, and filtered. The precipitate was recrystallized from methanol to afford compounds 2a, b and/or 3a, b respectively.

*6-(4-chloro-3-nitrophenyl)-4-(4-methoxyphenyl)-5*,*6-dihydropyrimidine-2(1 H)-thione (2a).*

From methanol (75%); mp 135–137 °C; IR ν cm^− 1^: 3223 (NH); 1172 (C = S); ^1^H-NMR (DMSO-d6, δ, ppm): 3.24 (d, 2 H, CH_2_), 3.62 (s, 3 H, OCH_3_), 4.23 (t, 1H, CH), 6.92–7.45 (m, 7 H, 7Haromatic), 9.24 (s, 1H, NH exchangeable with D2O); ^13^C-NMR (DMSO-d6, δ, ppm): 36.18, 57.14, 117.35, 122.54, 124.41, 129.13, 132.36, 134.27, 136.32, 138.22, 140.23, 145.34, 148.32, 151.21, 153.28, 156.23, 169.15 (17 C); MS: m/z (%): 375 (M^+^, 29) and 194 (100, base peak); Anal. calcd for C_17_H_14_ClN_3_O_3_S (375.65): C, 54.33; H, 3.75; N, 11.18; Found: C, 54.14; H, 3.53; N, 11.01.

#### 4-(4-methoxyphenyl)-6-(5-methylfuran-2-yl)-5,6-dihydropyrimidine-2(1 H)-thione (2b)

From methanol (82%); mp 175–177 °C; IR ν cm^− 1^: 3215 (NH); 1170 (C = S); 1 H-NMR (DMSO-d6, δ, ppm): 2.81 (s, 3 H, CH_3_), 3.12 (d, 2 H, CH_2_), 3.75 (s, 3 H, OCH_3_), 4.19 (t, 1 H, CH), 6.92–7.45 (m, 6 H, 4Haromatic, 2 H furan), 9.13 (s, 1 H, NH exchangeable with D2O); ^13^C-NMR (DMSO-d6, δ, ppm): 38.22, 52.37, 116.45, 121.71, 124.42, 129.31, 133.26, 134.18, 136.21, 139.24, 140.27, 144.35, 147.13, 154.28, 157.16, 171.23 (16 C); MS: m/z (%): 300 (M^+^, 35) and 208 (100, base peak); Anal. calcd for C_16_H_16_N_2_O_2_S (300.24): C, 63.98; H, 5.37; N, 9.33; Found: C, 63.76; H, 5.18; N, 9.12.

#### 6-(4-chloro-3-nitrophenyl)-4-(4-methoxyphenyl)-5,6-dihydropyrimidin-2(1 H)-one (3a)

From methanol (62%); mp 189–191 °C; IR ν cm^− 1^: 3225 (NH); 1752 (C = O); ^1^H-NMR (DMSO-d6, δ, ppm): 3.45 (s, 3 H, OCH_3_), 4.52 (d, 2 H, CH_2_), 5.16 (t, 1H, CH), 6.87–7.39 (m, 7 H, 7Haromatic), 9.62 (s, 1H, NH exchangeable with D_2_O); ^13^C-NMR (DMSO-d6, δ, ppm): 36.18, 57.14, 116.35, 121.54, 124.41, 128.13, 131.36, 134.27, 136.32, 139.22, 140.23, 145.34, 148.43, 151.36, 153.24, 156.15, 167.26 (17 C); MS: m/z (%): 359 (M^+^, 28) and 202 (100, base peak); Anal. calcd for C_17_H_14_ClN_3_O_4_ (359.25): C, 56.76; H, 3.92; N, 11.68; Found: C, 56.54; H, 3.71; N, 11.49.

#### 4-(4-methoxyphenyl)-6-(5-methylfuran-2-yl)-5,6-dihydropyrimidine-2(1 H)- one (3b)

From methanol (76%); mp 185–187 °C; IR ν cm^− 1^: 3294 (NH); 1750 (C = O); ^1^H-NMR (DMSO-d6, δ, ppm): 2.57 (s, 3 H, CH_3_), 3.82 (s, 3 H, OCH_3_), 3.47 (d, 2 H, CH_2_), 4.35 (t, 1H, CH), 6.95–7.38 (m, 6 H, 4Haromatic, 2 H furan), 10.21 (s, 1H, NH exchangeable with D_2_O); ^13^C-NMR (DMSO-d6, δ, ppm): 39.21, 54.37, 117.45, 122.71, 125.42, 129.31, 131.26, 133.18, 135.21, 138.37, 141.27, 146.26, 149.13, 155.28, 158.23, 169.46 (16 C); MS: m/z (%): 284 (M^+^, 36) and 214 (100, base peak); Anal. calcd for C_16_H_16_N_2_O_3_ (284.24): C, 67.59; H, 5.67; N, 9.85; Found: C, 67.38; H, 5.48; N, 9.63.

Synthesis of compounds 4a, b.

A mixture of the chalcone 1a and/or 1b respectively (0.19 mol) and hydrazine (0.19 mol) was heated under reflux for 7 h. in acetic acid (30 mL). Then the reaction mixture was cooled, filtered, dried, and recrystallized from proper solvent to afford compounds 4a and 4b respectively.

*1-(5-(4-chloro-3-nitrophenyl)-3-(4-methoxyphenyl)-4*,*5-dihydro-1H-pyrazol-1-yl)ethan-1-one (4a).* From methanol (67%); mp 234–236 °C; IR ν cm^− 1^: 1755 (C = O); ^1^H-NMR (DMSO-d6, δ, ppm): 2.83 (s, 3 H, CH_3_), 3.76 (s, 3 H, OCH_3_), 3.82 (d, 2 H, CH_2_), 4.92 (t, 1H, CH), 6.87–7.41 (m, 7 H, 7Haromatic); ^13^C-NMR (DMSO-d6, δ, ppm): 37.36, 58.24, 113.15, 119.45, 121.53, 125.26, 129.32, 131.29, 133.26, 135.34, 138.31, 141.24, 146.29, 149.13, 152.57, 158.34, 160.24, 163.13 (18 C); MS: m/z (%): 373 (M^+^, 31) and 188 (100, base peak); Anal. calcd for C_18_H_16_ClN_3_O_4_ (373.16): C, 57.84; H, 4.31; N, 11.24; Found: C, 57.65; H, 4.11; N, 11.05.

#### 1-(3-(4-methoxyphenyl)-5-(5-methylfuran-2-yl)-4,5-dihydro-1 H-pyrazol-1-yl)ethan-1-one (4b)

From ethanol (63%); mp 217–219 °C; IR ν cm^− 1^: 1752 (C = O); ^1^H-NMR (DMSO-d6, δ, ppm): 2.45 (s, 3 H, CH_3_), 3.26 (s, 3 H, CH_3_), 3.82 (s, 3 H, OCH_3_), 3.92 (d, 2 H, CH_2_), 5.19 (t, 1H, CH), 7.15–7.39 (m, 6 H, 6Haromatic); ^13^C-NMR (DMSO-d6, δ, ppm): 31.24, 35.28, 59.23, 114.17, 120.45, 122.31, 127.24, 129.32, 132.21, 134.34, 137.27, 141.35, 145.22, 148.13, 151.24, 157.25, 161.31 (17 C); MS: m/z (%): 298 (M^+^, 35) and 192 (100, base peak); Anal. calcd for C_17_H_18_N_2_O_3_ (298.32): C, 68.44; H, 6.08; N, 9.39; Found: C, 68.25; H, 5.86; N, 9.18.

Synthesis of compounds 5a, b.

A mixture of the chalcone 1a and/or 1b respectively (0.19 mol) and phenylhydrazine (0.19 mol) was heated under reflux for 10 h. in ethanol (20 mL). Then the reaction mixture was cooled, filtered, dried, and recrystallized from proper solvent to afford compounds 5a and 5b respectively.

#### 5-(4-chloro-3-nitrophenyl)-3-(4-methoxyphenyl)-1-phenyl-4,5-dihydro-1 H-pyrazole (5a)

From ethanol (61%); mp 196–198 °C; IR ν cm^− 1^: 1672 (C = N); 1566 (C = C); ^1^H-NMR (DMSO-d6, δ, ppm): 3.26 (s, 3 H, OCH_3_), 3.41 (d, 2 H, CH_2_), 4.87 (t, 1H, CH), 7.25–7.63 (m, 12 H, Haromatic); ^13^C-NMR (DMSO-d6, δ, ppm): 39.24, 55.25, 92.24, 113.23, 117.42, 119.28, 122.42, 125.15, 127.29, 129.19, 132.52, 135.26, 137.46, 139.21, 141.35, 145.52, 147.26, 149.27, 151.64, 153.19, 155.92, 158.14 (22 C); MS: m/z (%): 407 (M^+^, 43) and 252 (100, base peak); Anal. calcd for C_22_H_18_ClN_3_O_3_ (407.32): C, 64.79; H, 4.45; N, 10.30; Found: C, 64.58; H, 4.27; N, 10.11.

#### 3-(4-methoxyphenyl)-5-(5-methylfuran-2-yl)-1-phenyl-4,5-dihydro-1 H-pyrazole (5b)

From ethanol (69%); mp 267–269 °C; IR ν cm^− 1^: 1680 (C = N); 1565 (C = C); ^1^H-NMR (DMSO-d6, δ, ppm): 2.62 (s, 3 H, CH_3_), 3.43 (s, 3 H, OCH_3_), 4.69 (d, 2 H, CH_2_), 5.14 (t, 1H, CH), 7.12–7.49 (m, 11 H, 9Haromatic, 2Hfuran); ^13^C-NMR (DMSO-d6, δ, ppm): 26.33, 37.17, 56.28, 94.19, 113.25, 118.36, 121.24, 124.46, 126.21, 129.18, 132.52, 135.35, 137.46, 140.24, 142.32, 146.42, 149.53, 152.62, 155.27, 157.46, 159.24 (21 C); MS: m/z (%): 332 (M^+^, 33) and 212 (100, base peak); Anal. calcd for C_21_H_20_N_2_O_2_ (332.43): C, 75.88; H, 6.06; N, 8.43; Found: C, 75.69; H, 5.86; N, 8.24.

Synthesis of compounds 6a, b -9a, b.

A solution of the chalcone 1a and 1b respectively (0.19 mol) and active methylene reagents namely, ethylcyanoacetate, acetylacetone, malononitrile and/or ethylacetoacetate (0.19 mol) respectively in dry pyridine (15 mL) was refluxed for 3 h. The solution was cooled and poured onto ice/HCl and the solid that formed was filtered, washed several times with water, dried, and recrystallized from proper solvent to get of **6a**,** b -9a**,** b** respectively.

#### 4-(4-chloro-3-nitrophenyl)-2-hydroxy-6-(4-methoxyphenyl)-4 H-pyran-3-carbonitrile (6a)

From methanol (66%); mp 261–263 °C; IR ν cm^− 1^: 3314 (OH), 2223 (CN); ^1^H-NMR (DMSO-d6, δ, ppm): 3.24 (s, 3 H, OCH_3_), 3.83 (d, 1H, CH), 4.97 (d, 1H, CH), 6.89–7.46 (m, 7 H, Haromatic); 11.83 (s, 1H, OH exchangeable with D_2_O);13 C-NMR (DMSO-d6, δ, ppm): 28.36, 54.36, 93.24, 114.19, 117.43, 120.19, 123.46, 127.32, 129.27, 131.52, 134.26, 136.46, 139.17, 143.23, 148.15, 153.37, 156.28, 159.16, 168.23 (19 C); MS: m/z (%): 384 (M^+^, 29) and 215 (100, base peak); Anal. calcd for C_19_H_13_ClN_2_O_5_ (384.82): C, 59.31; H, 3.41; N, 7.28; Found: C, 59.12; H, 3.20; N, 7.07.

#### 2-hydroxy-6-(4-methoxyphenyl)-4-(5-methylfuran-2-yl)-4 H-pyran-3-carbonitrile (6b)

From ethanol (71%); mp 235–237 °C; IR ν cm^− 1^: 3328 (OH), 2221 (CN); ^1^H-NMR (DMSO-d6, δ, ppm): 2.52 (s, 3 H, CH_3_), 3.64 (s, 3 H, OCH_3_), 3.87 (d, 1H, CH), 4.96 (d, 1H, CH), 6.94–7.38 (m, 6 H, Haromatic), 11.28 (s, 1H, OH exchangeable with D_2_O); ^13^C-NMR (DMSO-d6, δ, ppm): 26.23, 32.17, 57.16, 112.31, 119.24, 122.19, 125.21, 128.34, 133.25, 135.18, 137.27, 139.12, 144.29, 149.24, 152.37, 155.23, 157.18, 164.25 (18 C); MS: m/z (%): 309 (M^+^, 36) and 204 (100, base peak); Anal. calcd for C_18_H_15_NO_4_ (309.34): C, 69.89; H, 4.89; N, 4.53; Found: C, 69.67; H, 4.68; N, 4.34.

#### 1-(4-(4-chloro-3-nitrophenyl)-6-(4-methoxyphenyl)-2-methyl-4 H-pyran-3-yl)ethan-1-one (7a)

From methanol (65%); mp 265–267 °C; IR ν cm^− 1^: 1723 (C = O); ^1^H-NMR (DMSO-d6, δ, ppm): 2.42 (s, 3 H, CH_3_), 2.65 (s, 3 H, CH_3_), 3.79 (s, 3 H, OCH_3_), 3.93 (d, 1H, CH), 5.15 (d, 1H, CH), 7.15–7.49 (m, 7 H, Haromatic); ^13^C-NMR (DMSO-d6, δ, ppm): 26.12, 31.24, 35.42, 112.25, 115.24, 116.19, 119.43, 120.19, 123.46, 127.32, 129.21, 131.36, 134.23, 136.28, 139.34, 145.23, 149.25, 153.26, 155.28, 158.24, 162.31 (21 C); MS: m/z (%): 399 (M^+^, 29) and 215 (100, base peak); Anal. calcd for C_21_H_18_ClNO_5_ (399.52): C, 63.09; H, 4.54; N, 3.50; Found: C, 62.87; H, 4.35; N, 3.31.

#### 1-(6-(4-methoxyphenyl)-2-methyl-4-(5-methylfuran-2-yl)-4 H-pyran-3-yl)ethan-1-one *(7b)*

From ethanol (67%); mp 240–242 °C; IR ν cm^− 1^: 1718 (C = O); ^1^H-NMR (DMSO-d6, δ, ppm): 2.31 (s, 3 H, CH_3_), 2.59 (s, 3 H, CH_3_), 2.89 (s, 3 H, CH_3_), 3.72 (s, 3 H, OCH_3_), 4.21 (d, 1H, CH), 5.13 (d, 1H, CH), 6.93–7.38 (m, 6 H, Haromatic); ^13^C-NMR (DMSO-d6, δ, ppm): 27.18, 31.45, 33.24, 36.36, 114.25, 119.32, 121.17, 124.28, 128.32, 132.25, 135.18, 137.27, 141.12, 147.29, 148.24, 152.37, 155.24, 157.25, 158.25, 162.42 (20 C); MS: m/z (%): 324 (M^+^, 32) and 192 (100, base peak); Anal. calcd for C_20_H_20_O_4_ (324.42): C, 74.06; H, 6.22; O, 19.73; Found: C, 73.85; H, 6.03; O, 19.56.

#### 2-amino-4-(4-chloro-3-nitrophenyl)-6-(4-methoxyphenyl)-4 H-pyran-3-carbonitrile (8a)

From methanol (75%); mp 210–212 °C; IR ν cm^− 1^: 3213 (NH_2_), 2225 (CN); ^1^H-NMR (DMSO-d6, δ, ppm): 3.19 (s, 3 H, OCH3), 3.47 (d, 1H, CH), 4.68 (d, 1H, CH), 7.25–7.63 (m, 7 H, Haromatic); 10.21 (s, 2 H, NH2 exchangeable with D2O); ^13^C-NMR (DMSO-d6, δ, ppm): 28.36, 54.36, 93.24, 114.19, 117.43, 120.19, 123.46, 127.32, 129.27, 131.52, 134.26, 136.46, 139.17, 143.23, 148.15, 153.37, 156.28, 159.16, 168.23 (19 C); MS: m/z (%): 383 (M^+^, 26) and 212 (100, base peak); Anal. calcd for C_19_H_14_ClN_3_O_4_ (383.15): C, 59.46; H, 3.68; N, 10.95; Found: C, 59.25; H, 3.49; N, 10.75.

#### 2-amino-6-(4-methoxyphenyl)-4-(5-methylfuran-2-yl)-4 H-pyran-3-carbonitrile (8b)

From ethanol (71%); mp 193–195 °C; IR ν cm^− 1^: 3218 (NH_2_), 2227 (CN); ^1^H-NMR (DMSO-d6, δ, ppm): 2.58 (s, 3 H, CH_3_), 3.46 (s, 3 H, O CH_3_), 3.82 (d, 1H, CH), 4.96 (d, 1H, CH), 6.92–7.47 (m, 6 H, Haromatic), 10.16 (s, 2 H, NH_2_ exchangeable with D_2_O); ^13^C-NMR (DMSO-d6, δ, ppm): 27.18, 31.45, 55.27, 91.31, 118.27, 121.19, 124.28, 128.32, 132.25, 135.18, 137.27, 139.12, 144.29, 149.24, 152.37, 155.23, 158.25, 169.13 (18 C); MS: m/z (%): 308 (M^+^, 24) and 202 (100, base peak); Anal. calcd for C_18_H_16_N_2_O_3_ (308.34): C, 70.12; H, 5.23; N, 9.09; Found: C, 69.93; H, 5.04; N, 8.89.

#### 1-(4-(4-chloro-3-nitrophenyl)-2-hydroxy-6-(4-methoxyphenyl)-4 H-pyran-3-yl)ethan-1-one (9a)

From ethanol (69%); mp 231–233 °C; IR ν cm^− 1^: 3315 (OH), 1718 (C = O); ^1^H-NMR (DMSO-d6, δ, ppm): 2.36 (s, 3 H, CH_3_), 3.72 (s, 3 H, O CH_3_), 4.26 (d, 1H, CH), 5.14 (d, 1H, CH), 7.17–7.58 (m, 7 H, Haromatic); 11.26 (s, 1H, OH exchangeable with D_2_O); ^13^C-NMR (DMSO-d6, δ, ppm): 28.36, 32.41, 93.24, 114.19, 117.43, 120.19, 123.46, 125.21, 127.32, 129.27, 131.52, 134.26, 136.46, 139.17, 143.23, 148.15, 153.37, 156.28, 159.16, 168.23 (20 C); MS: m/z (%): 401 (M^+^, 20) and 234 (100, base peak); Anal. calcd for C_20_H_16_ClNO_6_ (401.83): C, 59.79; H, 4.01; N, 3.49; Found: C, 59.57; H, 3.82; N, 3.28.

#### 1-(2-hydroxy-6-(4-methoxyphenyl)-4-(5-methylfuran-2-yl)-4 H-pyran-3-yl)ethan-1-one (9b)

From methanol (72%); mp 215–217 °C; IR ν cm^− 1^: 3388 (OH), 1718 (C = O); ^1^H-NMR (DMSO-d6, δ, ppm): 2.32 (s, 3 H, CH_3_), 2.41 (s, 3 H, CH_3_), 3.52 (s, 3 H, OCH_3_), 4.63 (d, 1H, CH), 5.18 (d, 1H, CH), 6.24–7.28 (m, 6 H, Haromatic), 9.32 (s, 1H, OH, exchangeable with D_2_O); ^13^C-NMR (DMSO-d6, δ, ppm): 25.12, 32.43, 56.24, 93.34, 116.27, 122.17, 124.28, 127.28, 129.31, 131.25, 135.14, 138.27, 139.18, 143.29, 148.24, 153.37, 155.25, 157.31, 168.14 (19 C); MS: m/z (%): 326 (M^+^, 32) and 204 (100, base peak); Anal. calcd for C_19_H_18_O_5_ (326.36): C, 69.93; H, 5.56; O, 24.51; Found: C, 69.74; H, 5.37; O, 24.32.

## Pharmacology activity

### Analgesic activity

Eight sets of 180 mice, each weighing between 20 and 25 g, were made, one for each sex. Eight sets of 180 mice of both sexes, each weighing 20–25 g, were created. The first group was given saline as a control, the second group was given gum acacia as a vehicle, and the third group was given Voltarene as a reference drug. The other groups were given SC administration (**2a**,** b-9a**,** b**). The mice were carefully placed in a 1 dm³ dry glass beaker that was maintained at a temperature of 55 to 56 0 C. We measured each mouse’s usual reaction time in seconds at 30, 60, 90, and 120-minute intervals. This is how long it takes the mouse to lick its feet or jamb after ingesting a dose of 5 mg/kg, starting from the moment it enters the warm beaker^37^.

### Anti-inflammatory activity

Evaluation 16 compounds **2a**,** b-9a**,** b** for their anti-inflammatory qualities. There were five sets of 65 adult albino rats (100–200 g) of each sex. The rate paw developed oedema when 0.1 cm3 of 20% Brewer’s yeast suspended in physiological saline solution was injected into the skin of the hind limb. In order to identify any inflammation brought on by the yeast, the paw’s thickness was assessed using a skin caliber after four hours. One group served as a control, while the other two groups were given intrapretenoal (I.P.) injections of flurbiprofen (20 mg/kg) and dimethyl sulfoxide (DMSO), respectively. The tested compounds were dissolved in DMSO and then given to the remaining groups at a concentration of 100 mg/kg. Scand measured the thickness of the paws three and six hours after injection^38^.

### System Preparation and molecular Docking

In preparation for molecular docking studies, the crystal structure of Human COX-2 (PDB: 6COX)^39^ was prepared with UCSF Chimera^40^. The system’s pH was optimized to 7.5 using PROPKA^41^. The 2D structure of the ligand was generated in ChemBioDraw Ultra 12.1^42^ and energetically minimized with the MMFF94 force field via a steepest descent approach in Avogadro^43^. Hydrogen atoms were then removed from the protein structure using UCSF Chimera^40^.

### Molecular Docking

Docking simulations were performed using AutoDock Vina^44^. Ligands were prepared with Gasteiger partial charges^45^ and AutoDock atom types^46^ assigned via the MGL tools interface. A grid box of 20 Å was centered at coordinates (24.44, 21.59, 45.69) on the 6COX structure, with an exhaustiveness value set to 8. The Lamarckian genetic algorithm^47^ was used to produce docked poses, which were sorted in descending order of docking score.

### Molecular dynamic (MD) simulations

Molecular dynamic (MD) simulations provide unique insights into the physical motions of atoms and molecules within biological systems, offering an invaluable perspective on dynamic processes like conformational changes and binding events^48^. All MD simulations were performed using the GPU-accelerated PMEMD engine in the AMBER 18 package^49^. Ligand partial charges were derived using the General Amber Force Field (GAFF) via the ANTECHAMBER tool^50^. Each system was prepared using the Leap module, solvated in a TIP3P water box with a 10 Å buffer, and neutralized with Na+/Cl- counterions. Energy minimization involved a two-step process: first, a 2000-step minimization with 500 kcal/mol restraints on the solute, followed by a 1000-step full minimization without restraints using the conjugate gradient algorithm.

The systems were then gradually heated from 0 to 300 K over 500 ps under a 10 kcal/mol harmonic restraint with a 1 ps collision frequency, maintaining constant volume and atom count. This was followed by a 500 ps equilibration period at 300 K. Finally, a 20 ns production simulation was conducted in the NPT ensemble (1 bar, 300 K) using the Berendsen barostat^51^. The simulations employed a 2 fs timestep, the SHAKE algorithm to constrain hydrogen bonds, the SPFP precision model, and a Langevin thermostat with a 1 ps collision frequency.

### Post-MD analysis

Following the molecular dynamics simulations, trajectories were saved at 1 ps intervals for subsequent analysis. The CPPTRAJ module^52^ within the AMBER18 suite was employed to process these trajectories. All resultant graphs and visualizations were generated using the Origin^53^ software package and UCSF Chimera^40^.

### Thermodynamic calculation

The Molecular Mechanics with Generalized Born and Surface Area solvation (MM/GBSA) and Poisson-Boltzmann Surface Area (MM/PBSA) methods are established computational approaches for estimating ligand-binding affinities^54^. These methods utilize trajectories from molecular simulations to calculate binding free energies within a rigorous statistical-mechanical framework for a given force field. For this study, the binding free energy was calculated as an average from 200 snapshots extracted across the 20 ns simulation trajectory. The change in binding free energy (ΔG) for the complex, ligand, and receptor is given by the following Eq. 5^6^:

1 $$\:\varDelta\:{\text{G}}_{\text{b}\text{i}\text{n}\text{d}}={\text{G}}_{\text{c}\text{o}\text{m}\text{p}\text{l}\text{e}\text{x}}-{\text{G}}_{\text{r}\text{e}\text{c}\text{e}\text{p}\text{t}\text{o}\text{r}}-{\text{G}}_{\text{l}\text{i}\text{g}\text{a}\text{n}\text{d}}$$

2 $$\:\varDelta\:{\text{G}}_{\text{b}\text{i}\text{n}\text{d}}={\text{E}}_{\text{g}\text{a}\text{s}}+{\text{G}}_{\text{s}\text{o}\text{l}}-\text{T}\text{S}\:$$

3 $$\:{\text{E}}_{\text{g}\text{a}\text{s}}={\text{E}}_{\text{i}\text{n}\text{t}}+{\text{E}}_{\text{v}\text{d}\text{w}}+{\text{E}}_{\text{e}\text{l}\text{e}}\:$$

4 $$\:{\text{G}}_{\text{s}\text{o}\text{l}}={\text{G}}_{\text{G}\text{B}}+{\text{G}}_{\text{S}\text{A}}$$

5 $$\:{\text{G}}_{\text{S}\text{A}}={\upgamma\:}\text{S}\text{A}\text{S}\text{A}\:$$

The gas-phase energy (Egas), composed of internal (Eint), Coulombic (Eele), and van der Waals (Evdw) components, was calculated directly using the FF14SB force field. The solvation free energy (Gsol) comprises polar (GGB) and non-polar (GSA) contributions. The non-polar component (GSA) was derived from the Solvent Accessible Surface Area (SASA)^56^ with a 1.4 Å water probe radius, while the polar component (GGB) was obtained by solving the Generalized Born (GB) equation. In the free energy equation, S represents the total entropy of the solute and T the temperature. Finally, the contribution of individual residues to the total binding free energy was determined using the MM/GBSA method implemented in Amber18.

## Conclusion

Chalcones **1a**,** b** were produced by reacting 4-methoxyacetophenone with aromatic aldehyde, namely 3-nitro-4-chlorobenzaldehyde and/or 5-methylfurfural in an alkaline medium respectively. Reaction of chalcone **1a**,** b** with thiourea and/or urea respectively afforded pyrimidinethione derivatives **2a**,** b** and **3a**,** b** respectively. Also, compound **1a**,** b** reacted with hydrazine in acetic acid and/or phenylhydrazine to afford pyrazolo derivatives **4a**,** b** and **5a**,** b** respectively. Reaction of compound **1a**,** b** with methylene reagents namely, ethylcyanoacetate, acetylacetone, malononitrile and/or ethylacetoacetate respectively afforded pyrane derivatives **6a**,** b-9a**,** b** respectively. The synthesis of thienopyrimidino derivatives **2a**,** b-9a**,** b** was conducted out using the starting material **1a**,** b**. Compound **1a**,** b** produced the corresponding pyrimidinethione derivatives **2a**,** b** and **3a**,** b** respectively through a reaction with thiourea and/or urea respectively. Also, compound **1a**,** b** reacted with hydrazine and/or phenylhydrazine to afford pyrazolo derivatives **4a**,** b** and **5a**,** b** respectively. Reaction of compound **1a**,** b** with methylene reagents namely, ethylcyanoacetate, acetylacetone, malononitrile and/or ethylacetoacetate respectively afforded pyrane derivatives **6a**,** b − 9a**,** b** respectively. Newly synthesized derivatives **2a**,** b-9a**,** b** have demonstrated pharmacological screening as having analgesic and anti-inflammatory properties on par with reference medications. All compounds tested were found to exhibit analgesic, where compounds **6b**,** 7b**,** 8b** and **9b** possessed higher significant analgesic. Also, all compounds tested were found to exhibit anti-inflammatory activities, where compounds **7b**,** 8a** and **9b** possessed higher significant anti-inflammatory activities. Also, molecular docking, molecular dynamic (MD) simulations and thermodynamic calculation were studied.

## Supplementary Information

Below is the link to the electronic supplementary material.

Supplementary Material 1

## Data Availability

All data generated or analyzed are included in this published article and its supplementary information file.
