# Supplementary material for: Pharmacological evaluation as analgesic and anti-inflammatory and molecular docking of newly synthesized nitrogen heterocyclic derivatives
Source: Sci Rep. 2025 Dec 20;15:44309. doi: 10.1038/s41598-025-31238-0 (PMC12722756; doi:10.1038/s41598-025-31238-0)

**Spectra of synthesized Compounds**

**
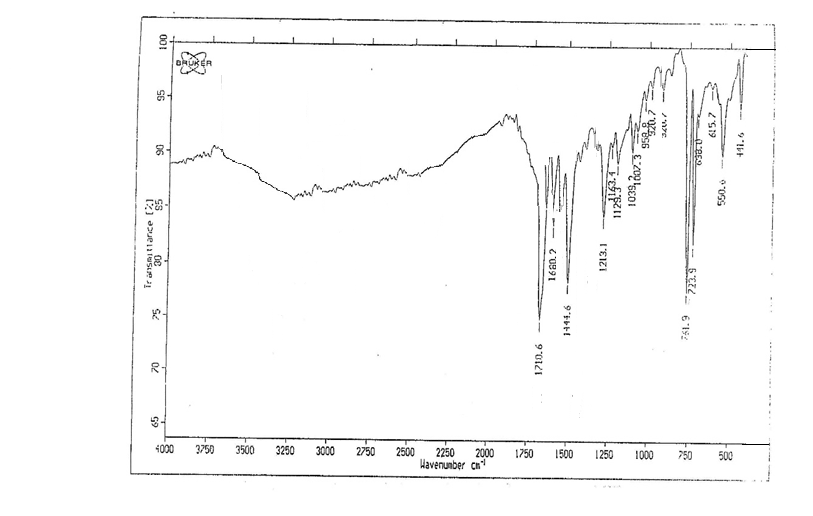
**

IR Spectra of Compound 1a

**
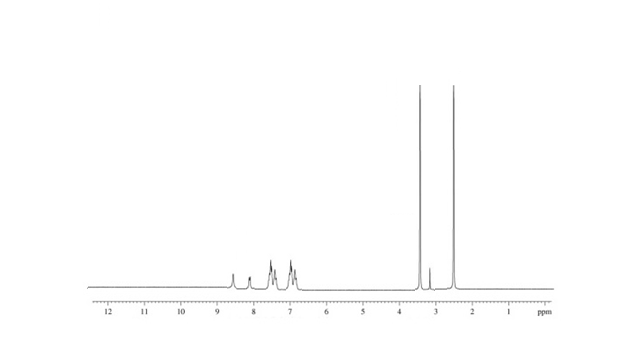
**

^1^H-NMR Spectra of Compound 1a


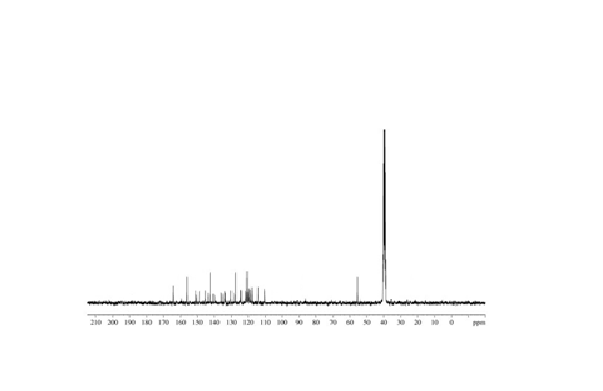


^13^C-NMR Spectra of Compound 1a


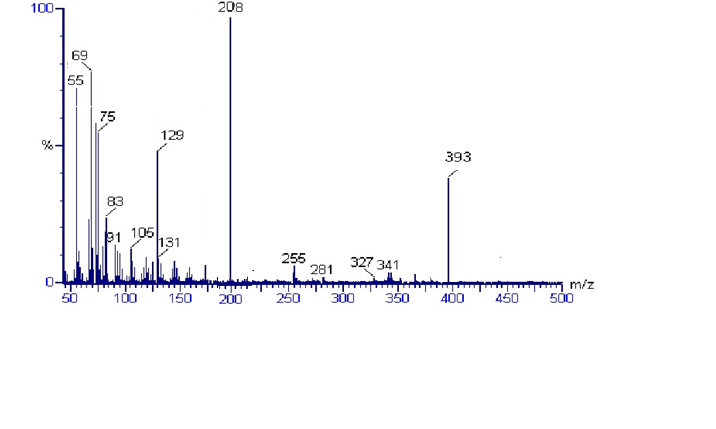


Maas Spectra of Compound 1a


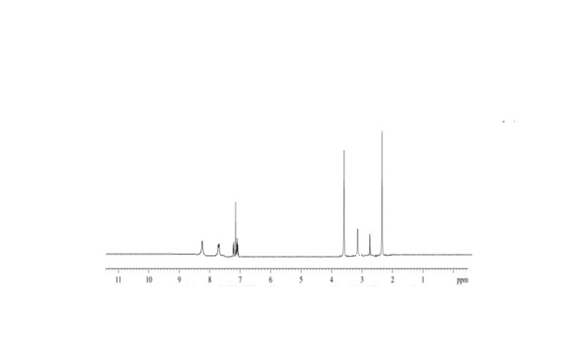


**^1^H-NMR Spectra of Compound 1b**

**
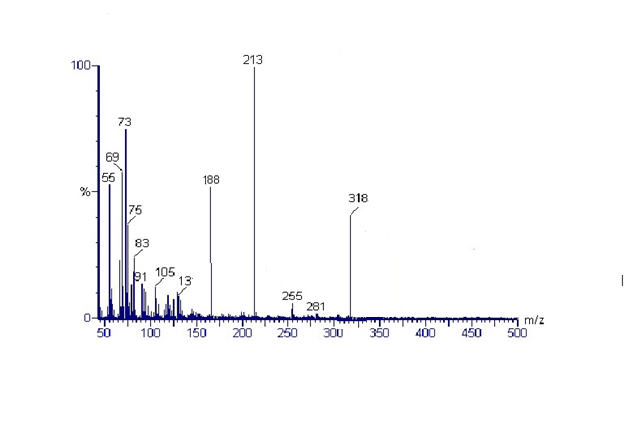
**

Maas Spectra of Compound 1b


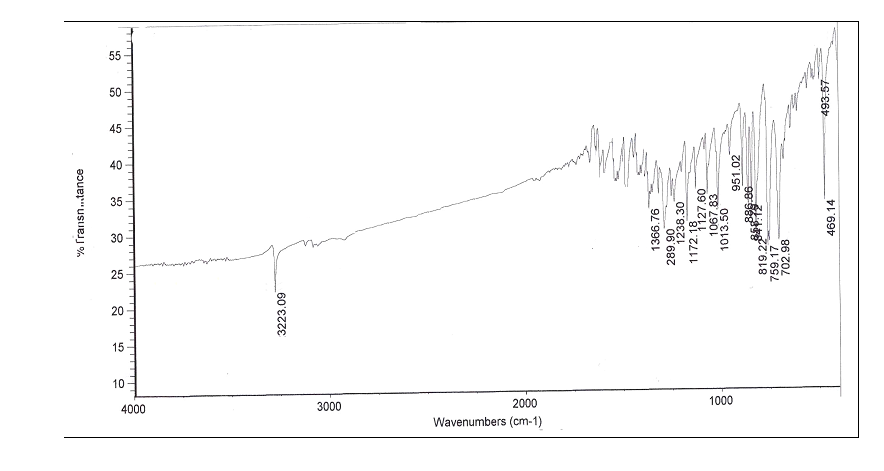


IR Spectra of Compound 2a


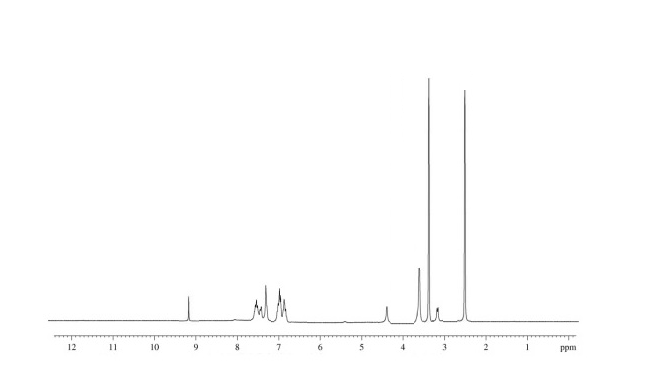


^1^H-NMR Spectra of Compound 2a


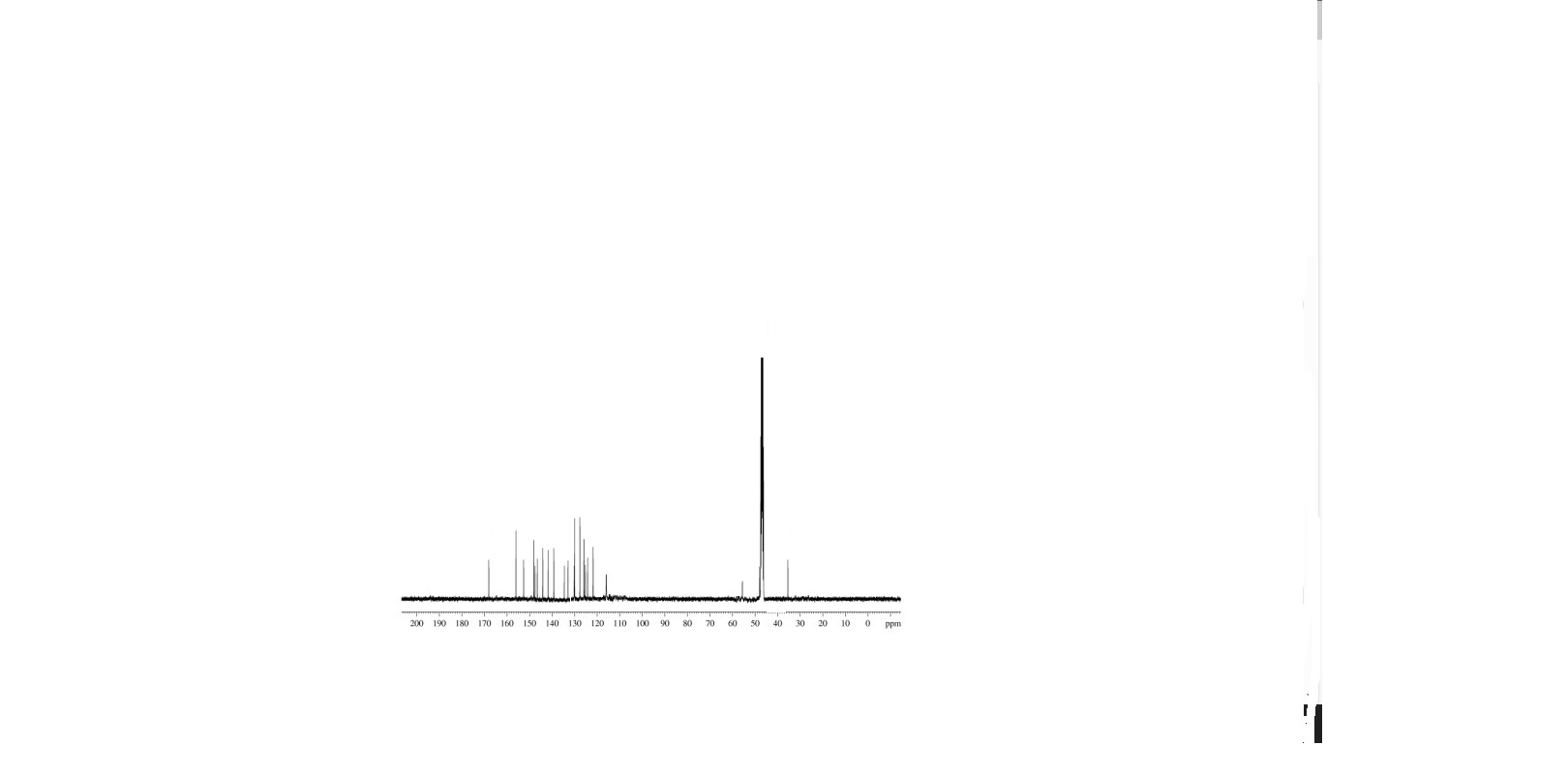


^13^C-NMR Spectra of Compound 2a


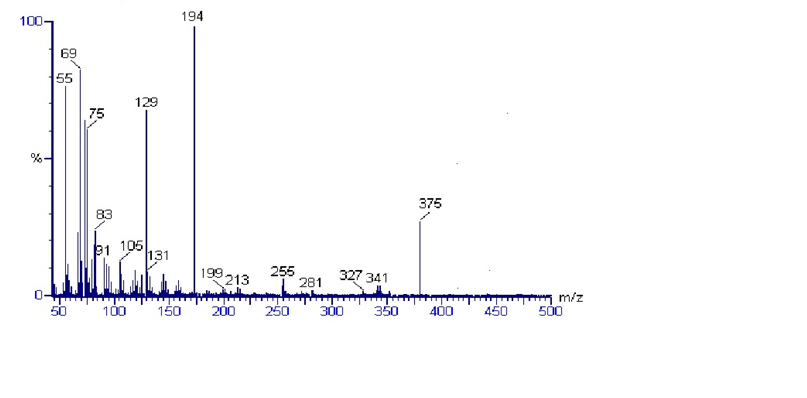


Maas Spectra of Compound 2a


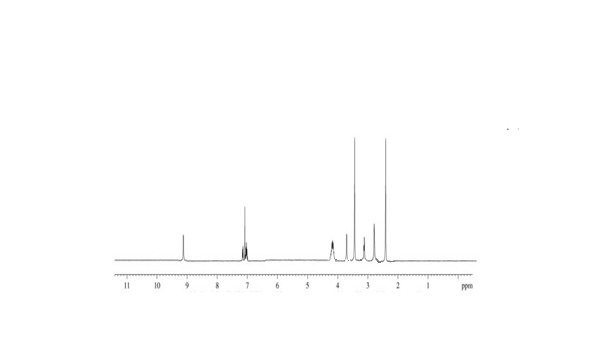


^1^H-NMR Spectra of Compound 2b


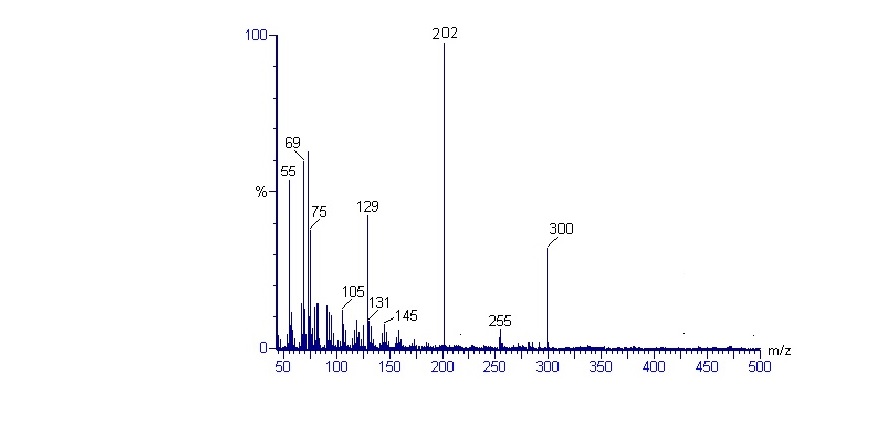


Mass Spectra of Compound 2b


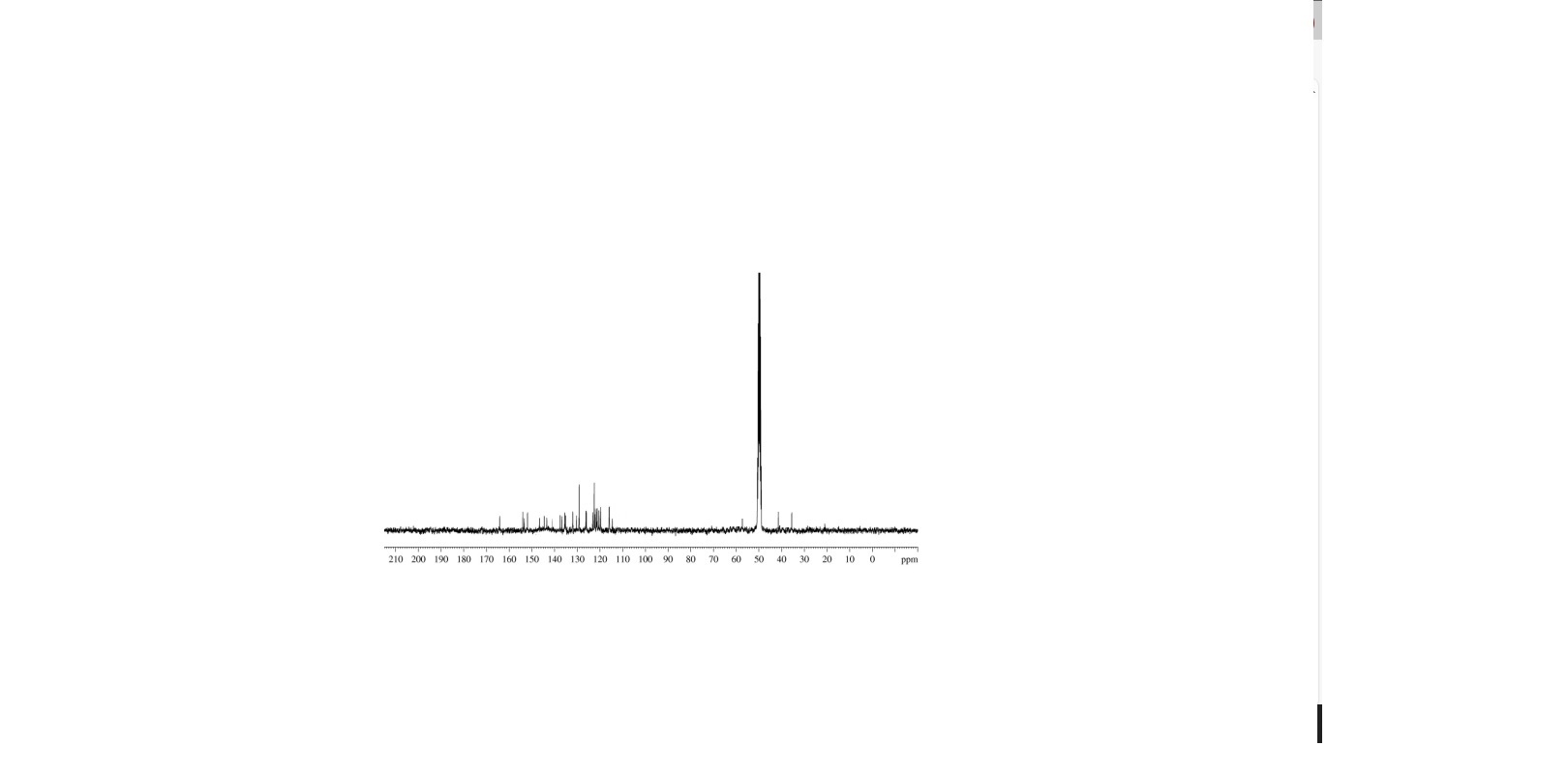


^13^C-NMR Spectra of Compound 3a


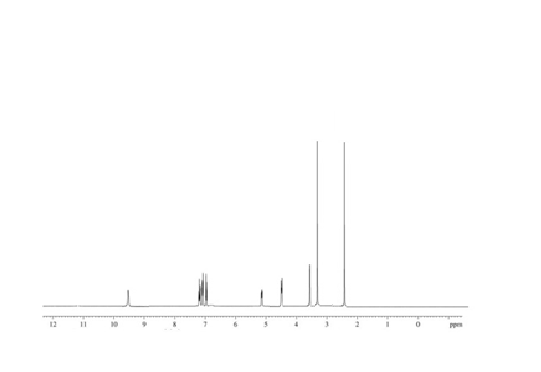


^1^H-NMR Spectra of Compound 3a


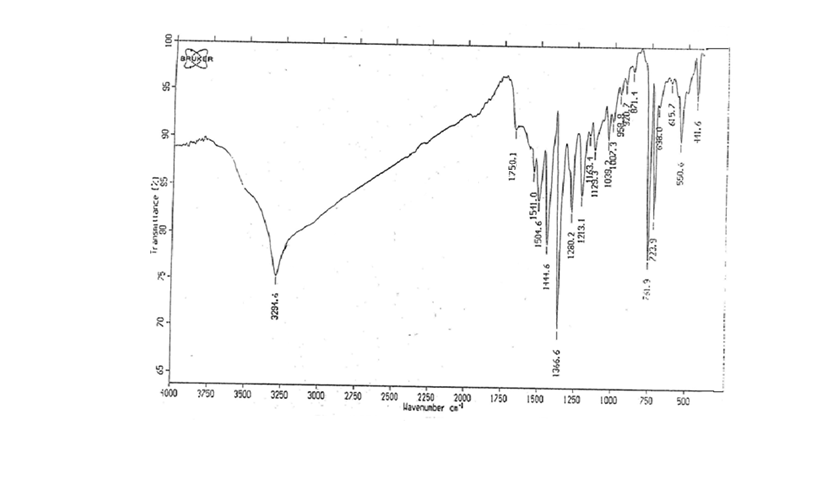


IR Spectra of Compound 3b


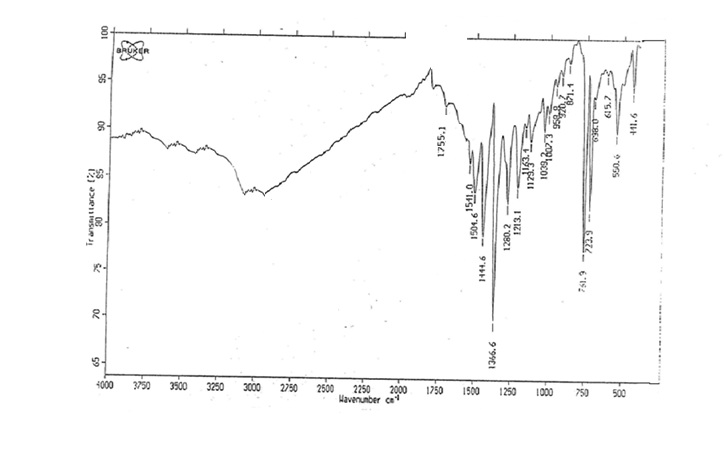


IR Spectra of Compound 4a


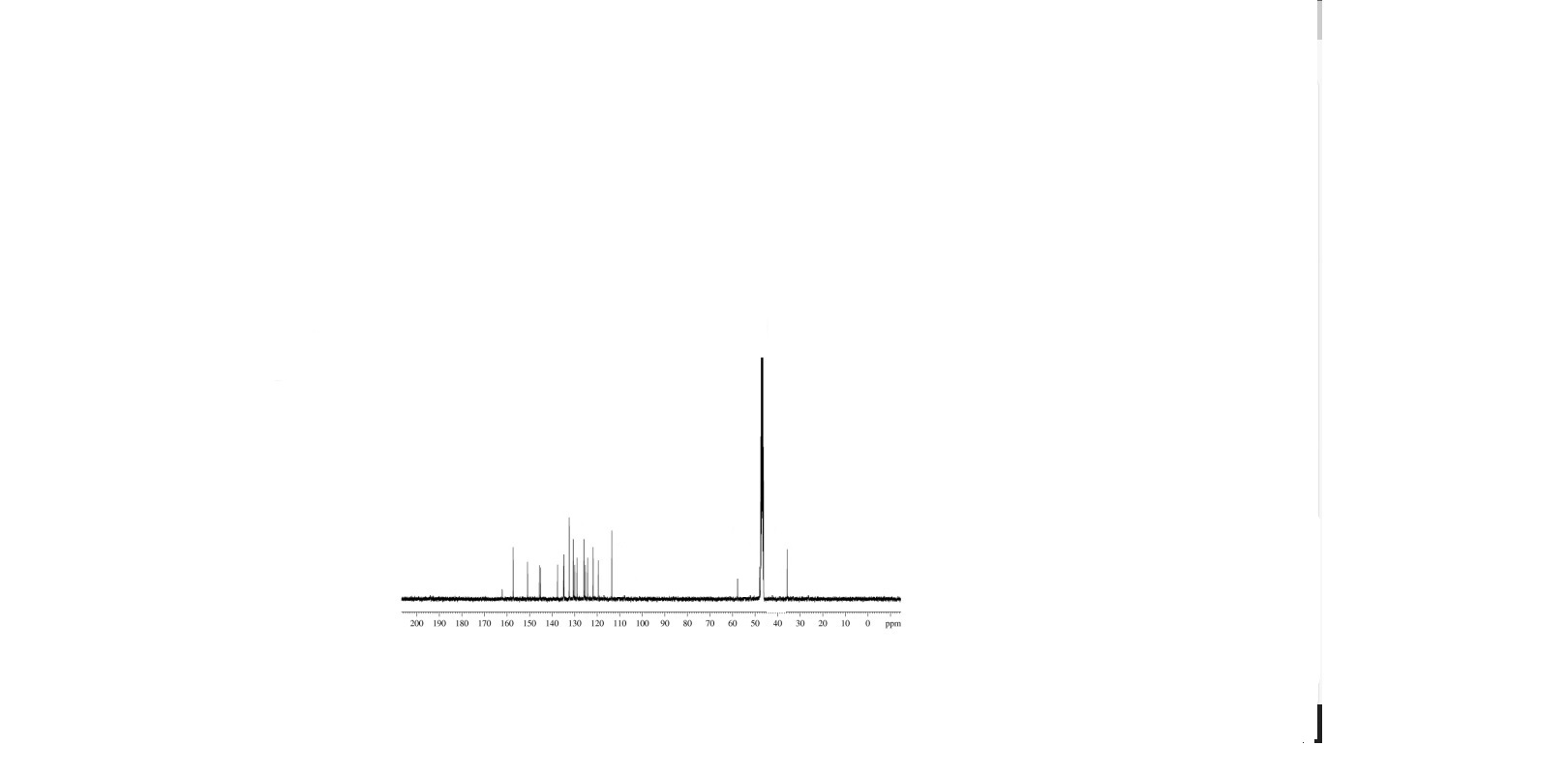


^13^C-NMR Spectra of Compound 4a


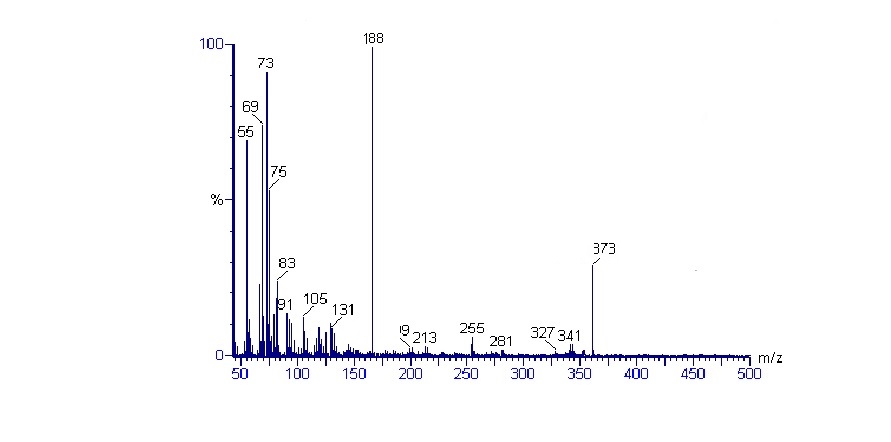


Mass Spectra of Compound 4a


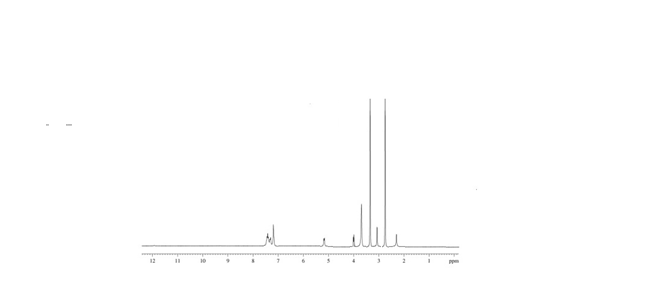


^1^H-NMR Spectra of Compound 4b


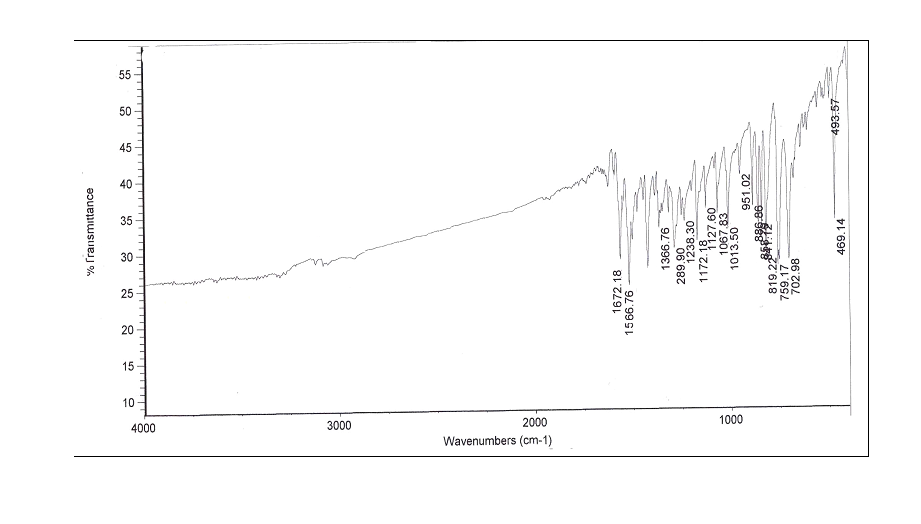


IR Spectra of Compound 5a


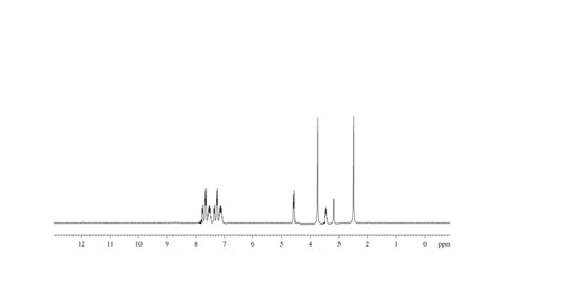


^1^H-NMR Spectra of Compound 5a


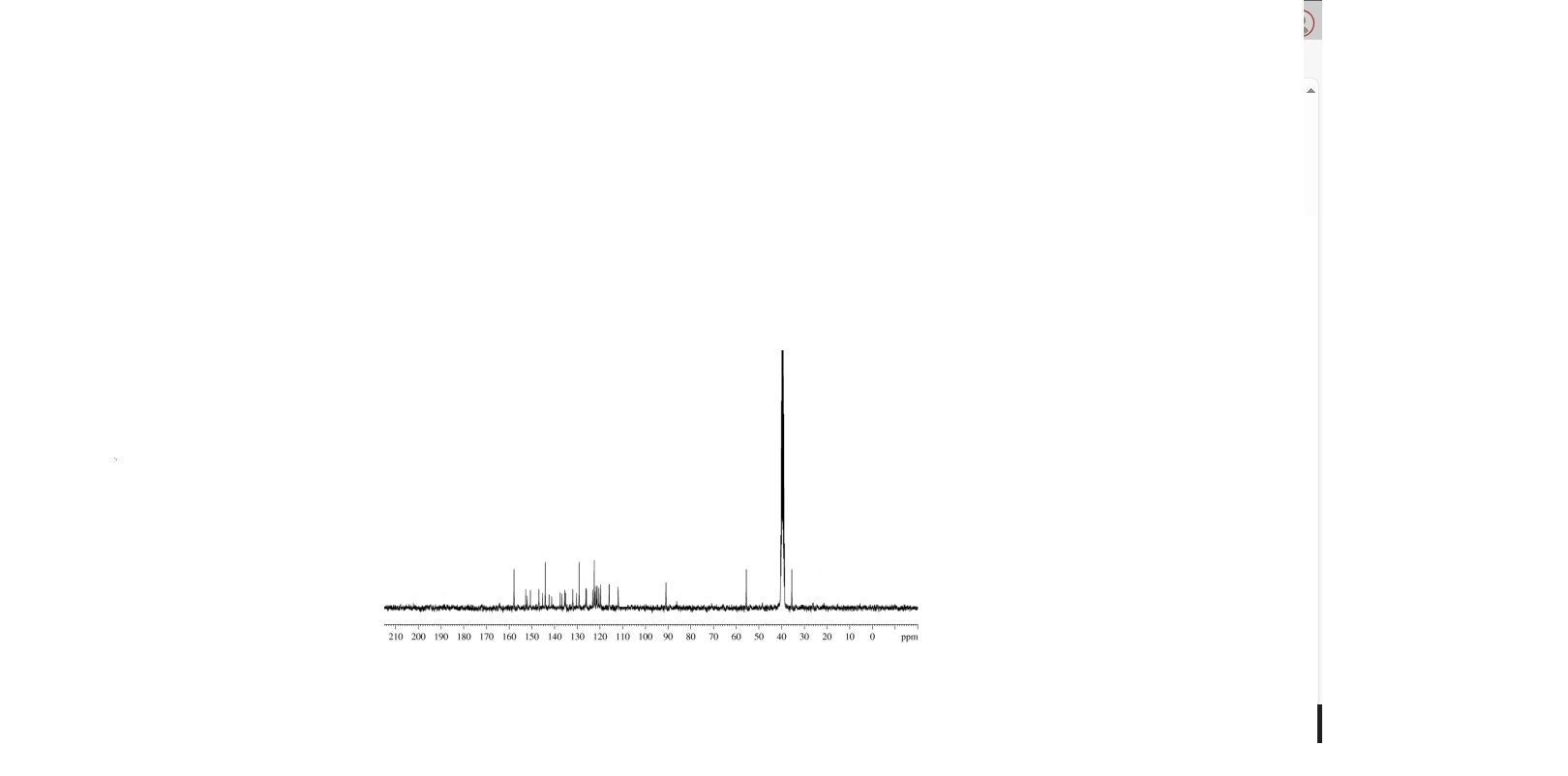


^13^C-NMR Spectra of Compound 5a


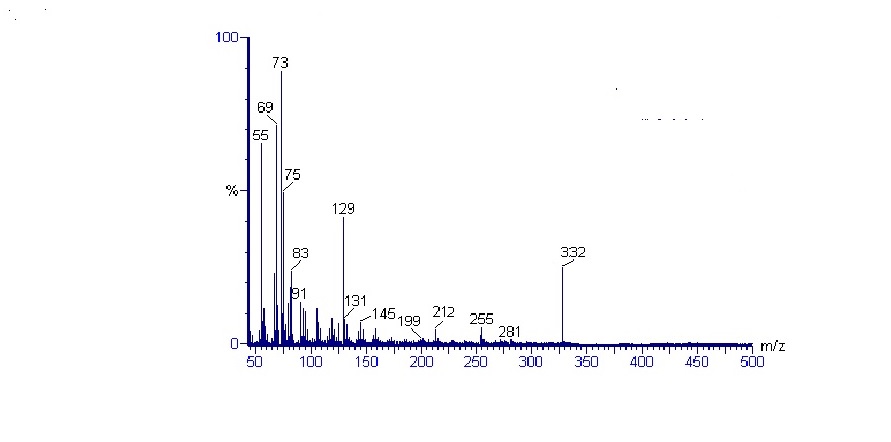


Mass Spectra of Compound 5b


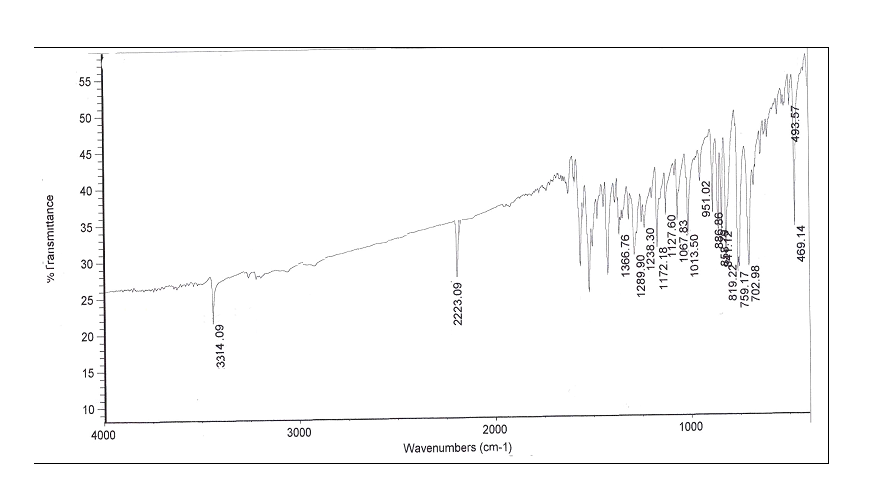


IR Spectra of Compound 6a


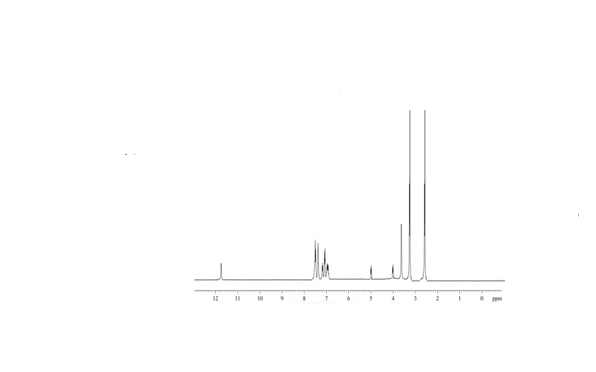


^1^H-NMR Spectra of Compound 6a


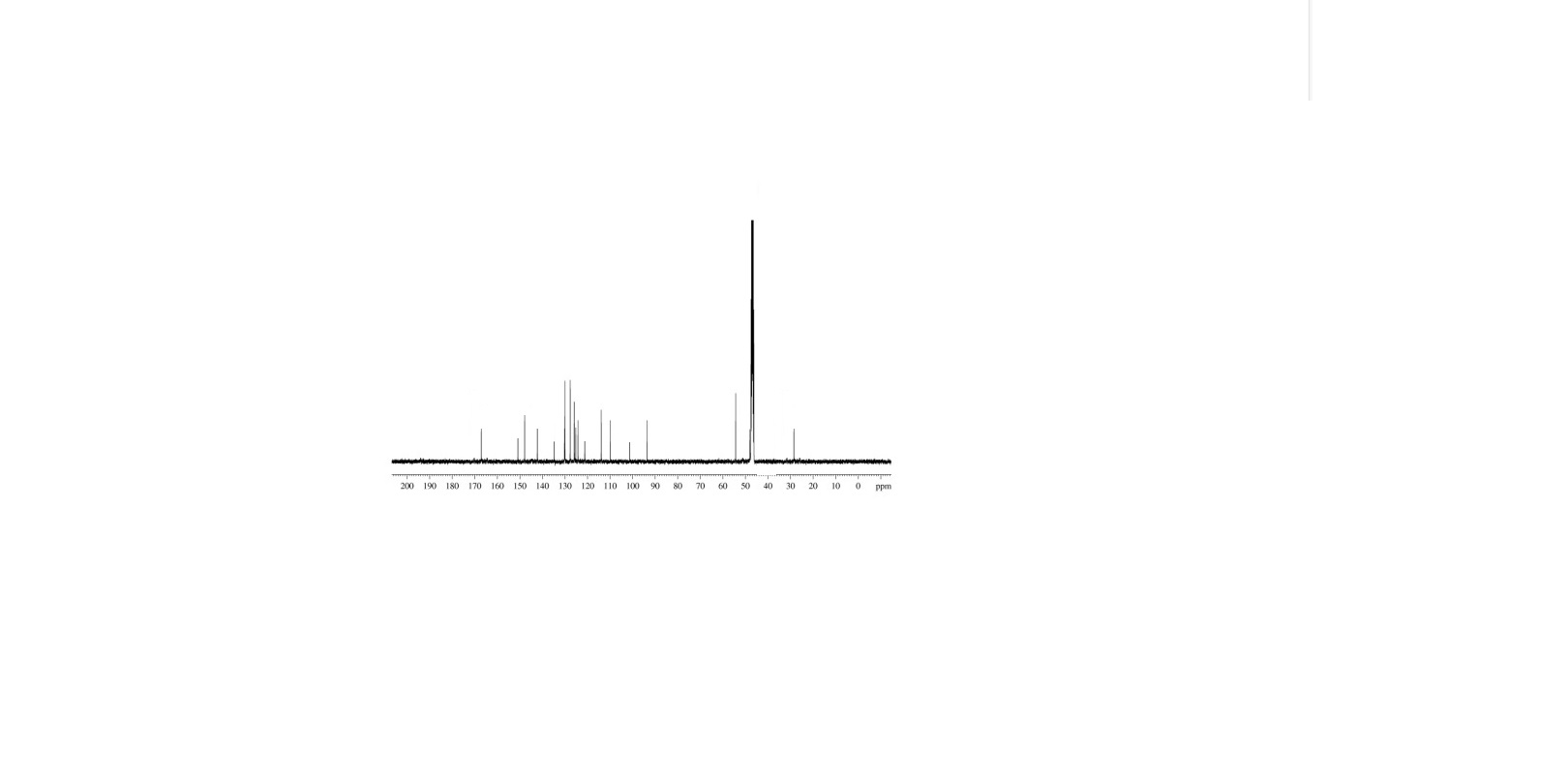


^13^C-NMR Spectra of Compound 6a


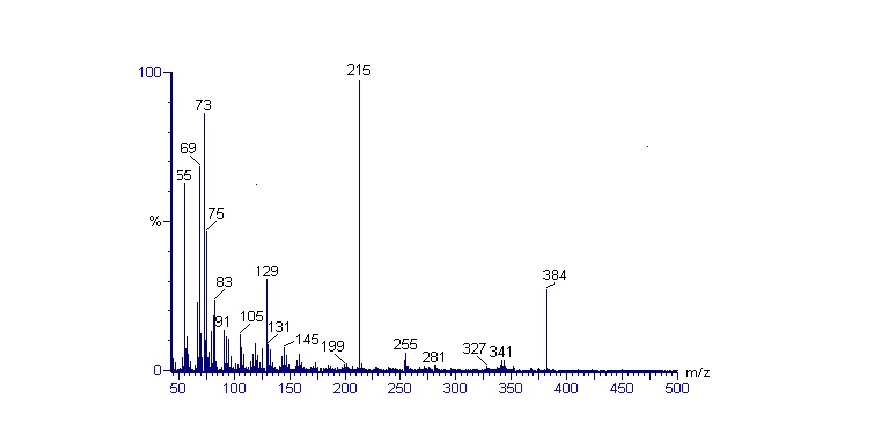


Mass Spectra of Compound 6a


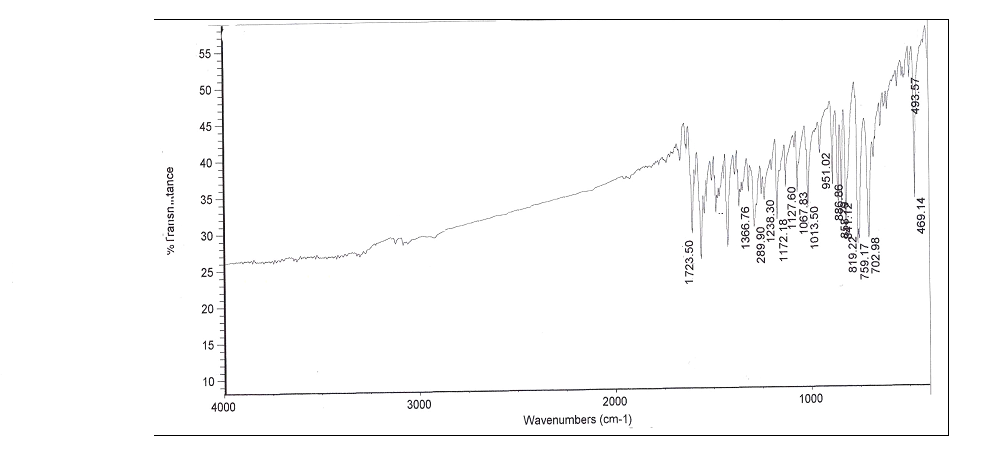


IR Spectra of Compound 7a


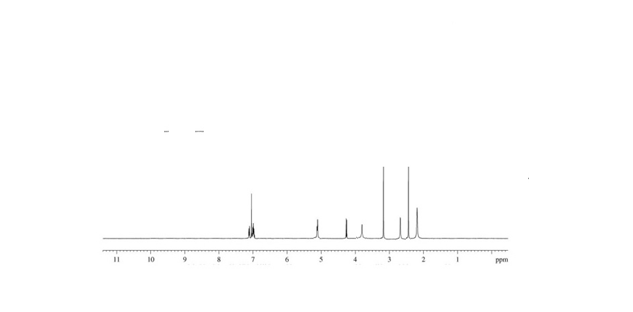


^1^H-NMR Spectra of Compound 7b


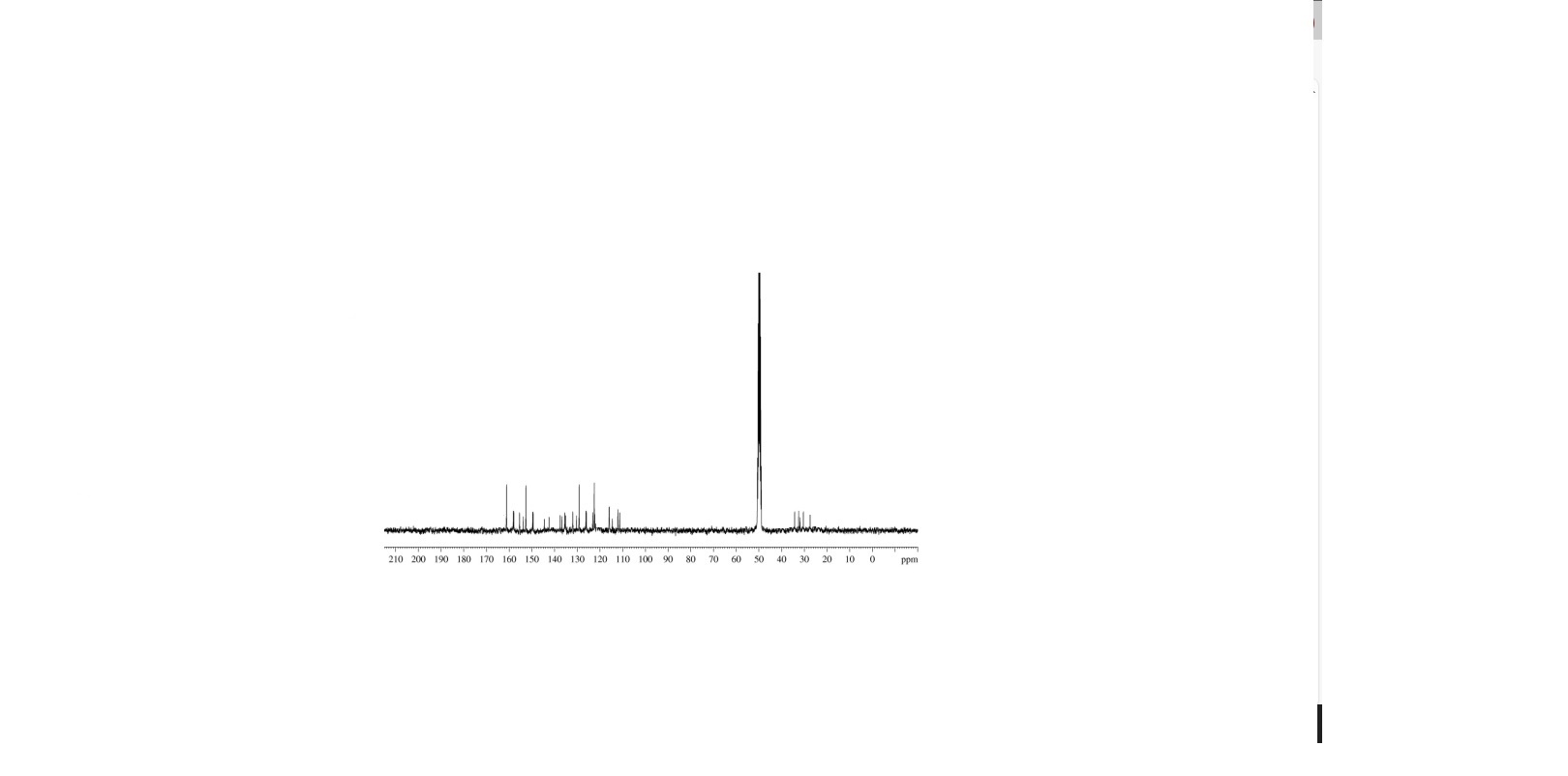


^13^C-NMR Spectra of Compound 7b


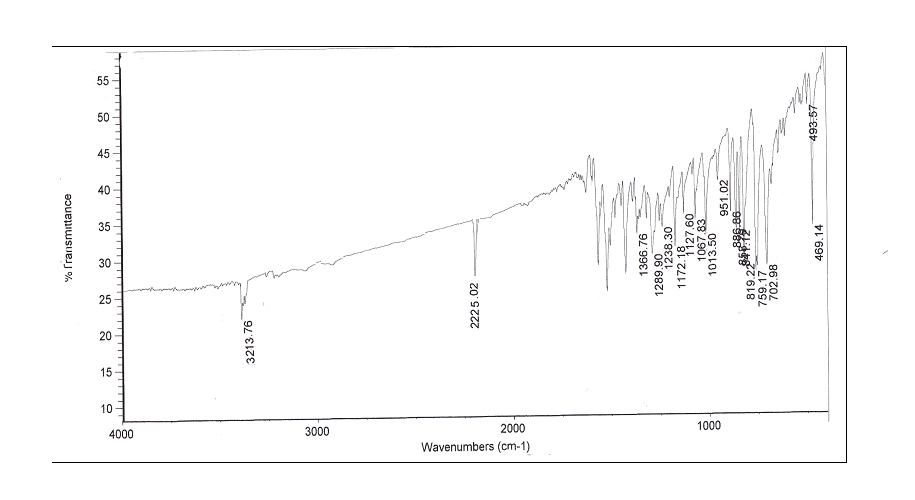


IR Spectra of Compound 8a


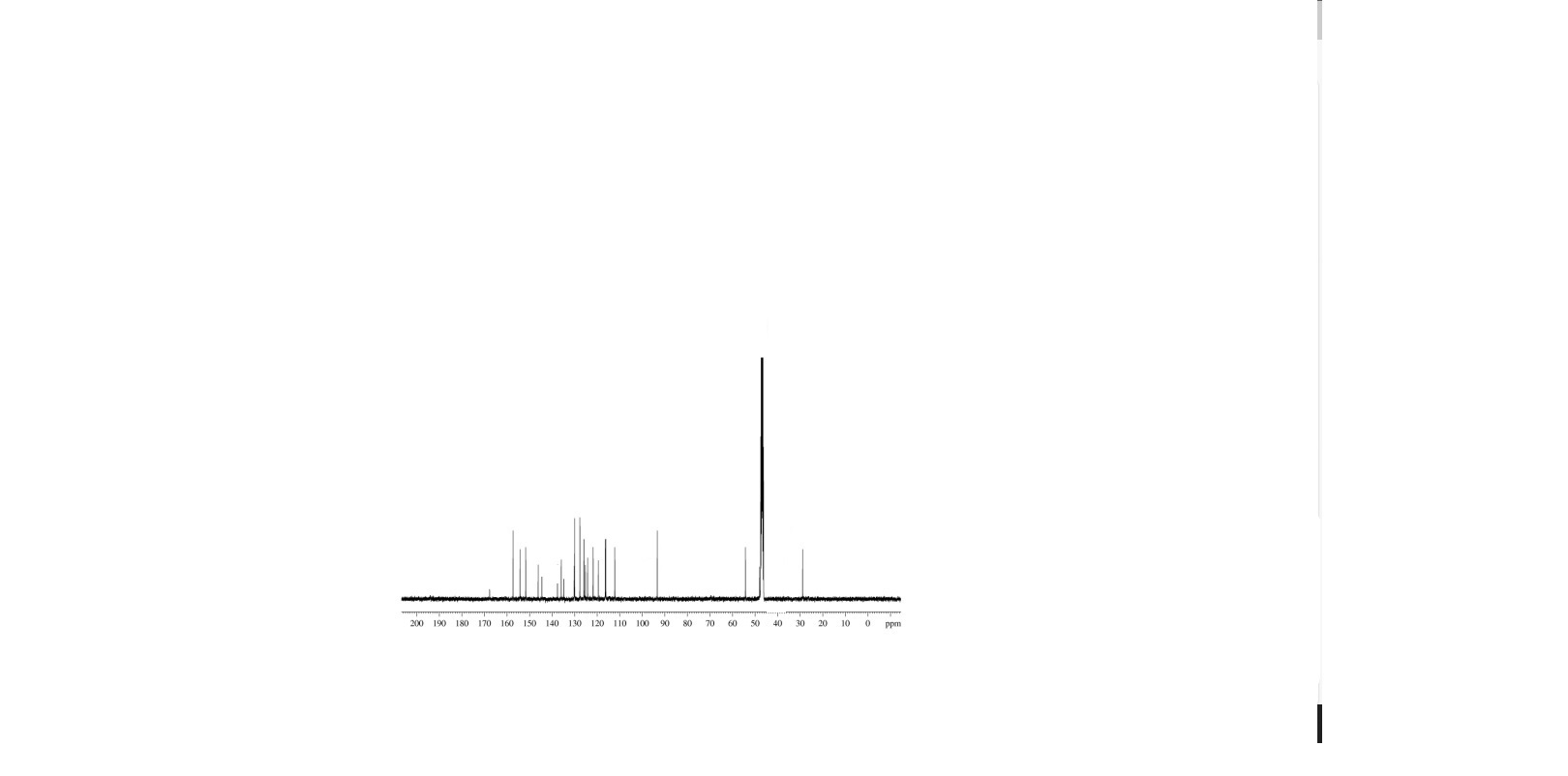


^13^C-NMR Spectra of Compound 8a


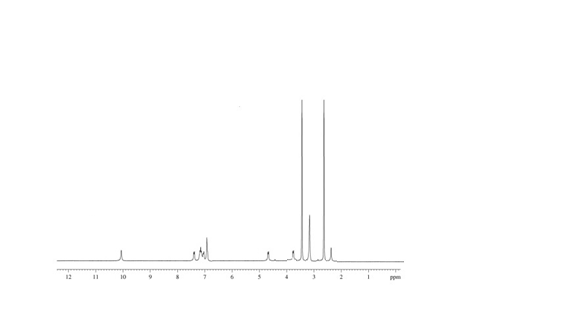


^1^H-NMR Spectra of Compound 8b


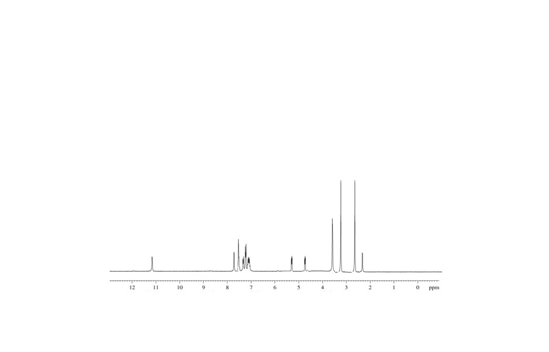


^1^H-NMR Spectra of Compound 9a


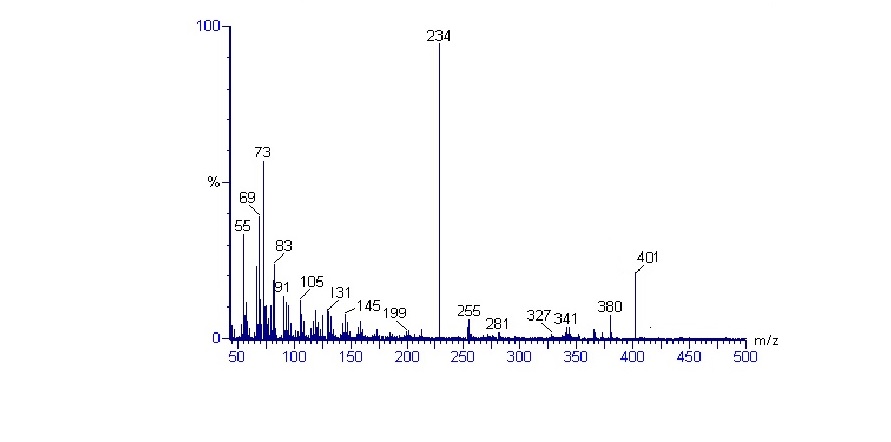


Mass Spectra of Compound 9a


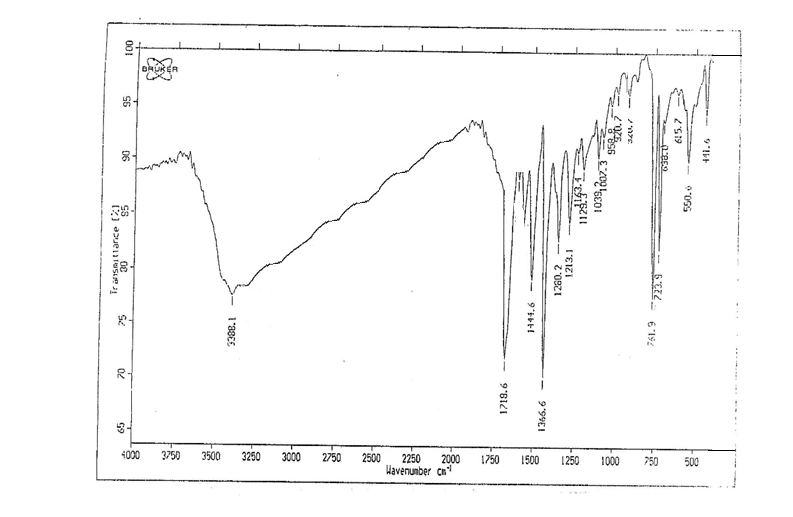


IR Spectra of Compound 9b


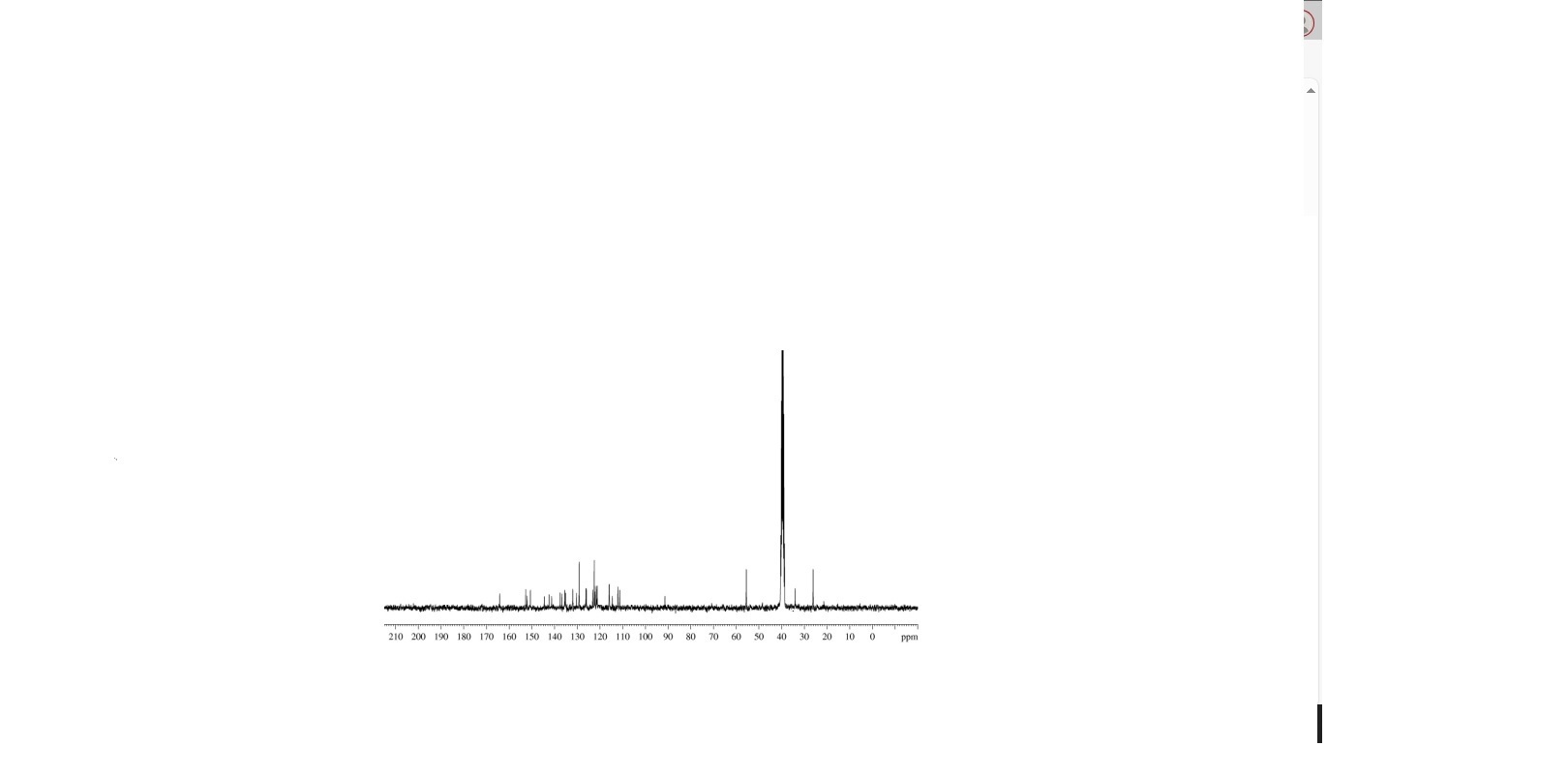


^13^C-NMR Spectra of Compound 9b

| Table S1: Molecular visualization of synthesized compound s at the binding site residue of human cyclooxygenase-2 (COX-2) receptor. | |
| --- | --- |
| 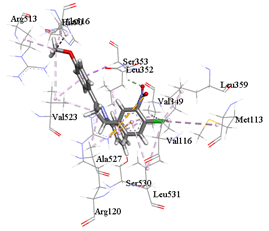 | 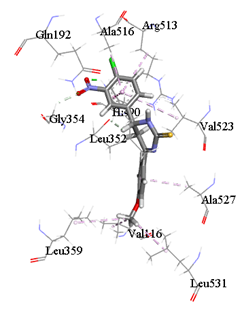 |
| 2a | 2b |
| 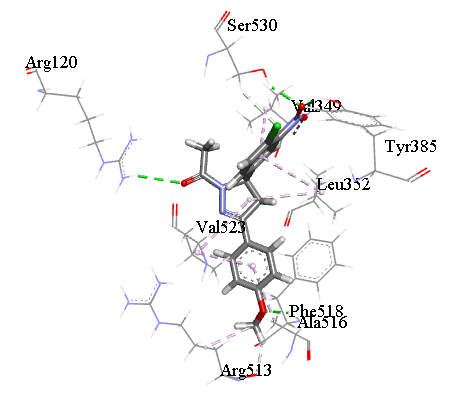 | 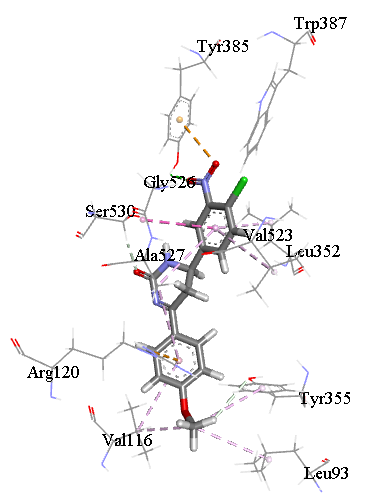 |
| 3a | 3b |
| 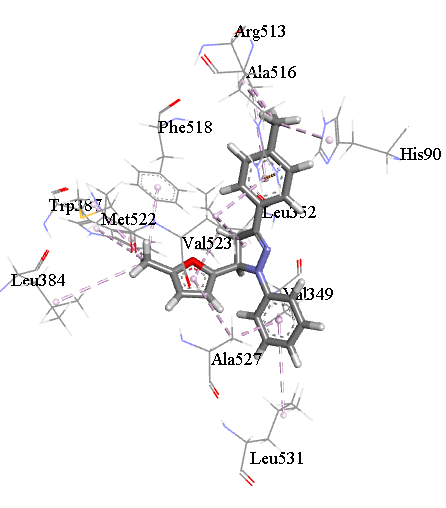 | 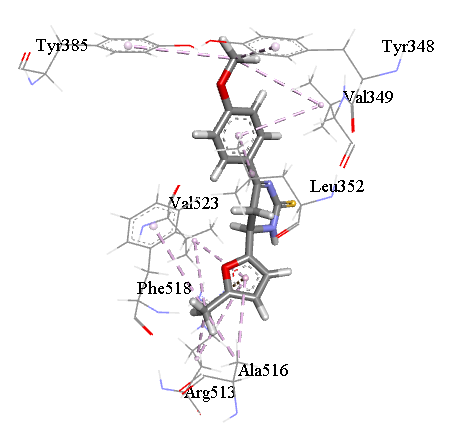 |
| 4a | 4b |
| 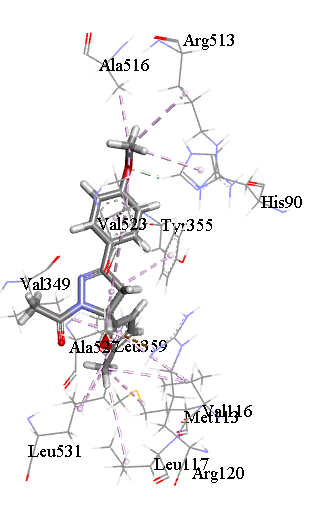 | 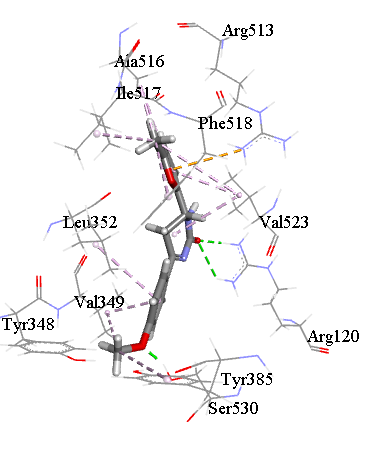 |
| 5a | 5b |
| 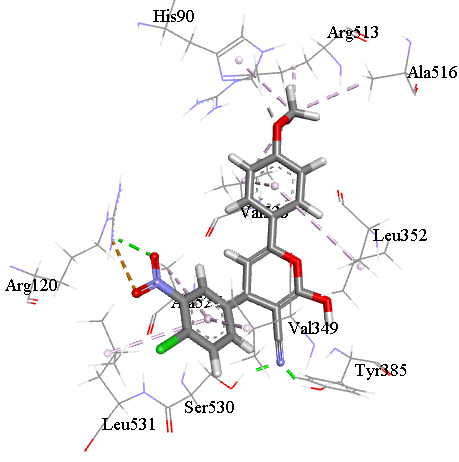 | 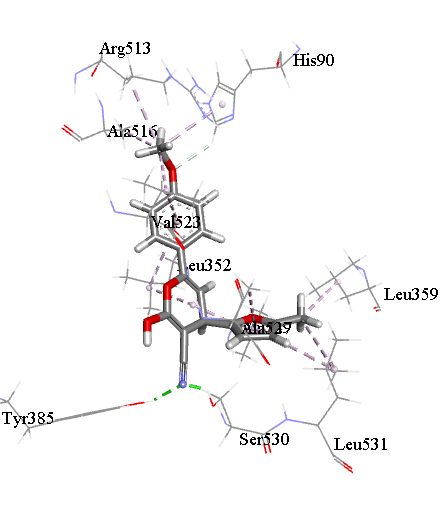 |
| 6a | 6b |
| 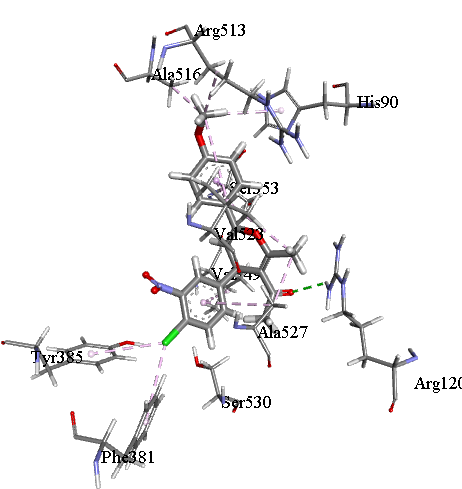 | 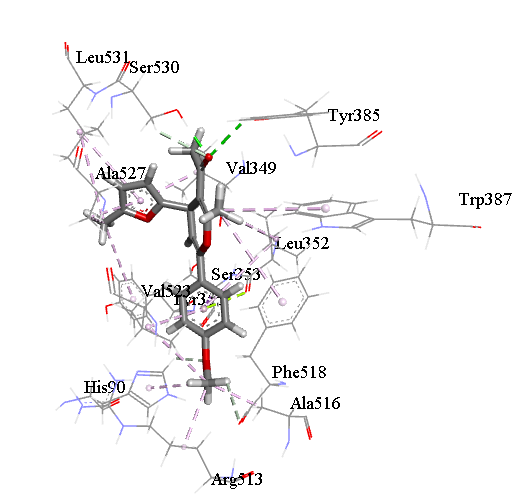 |
| 7a | 7b |
| 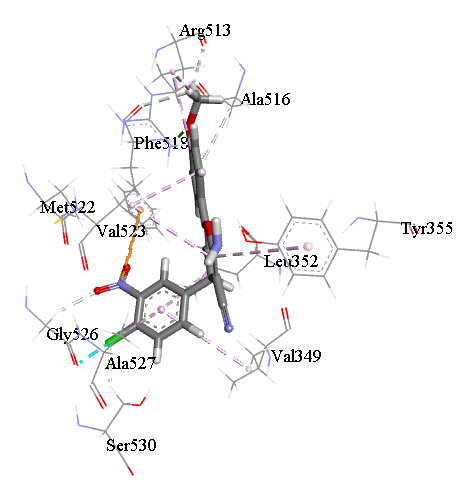 | 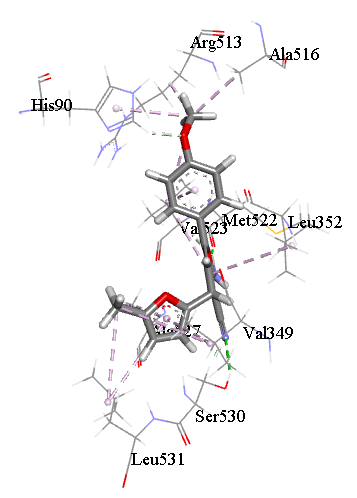 |
| 8a | 8b |
| *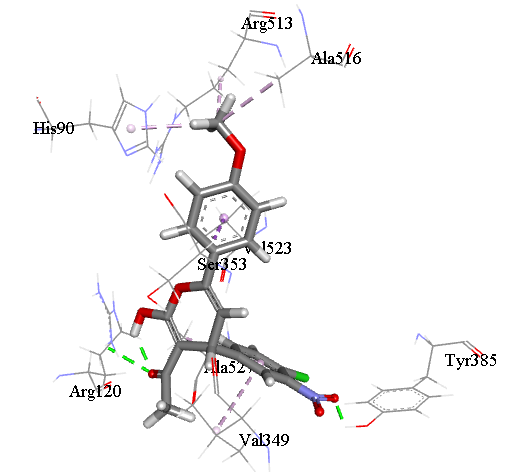* | 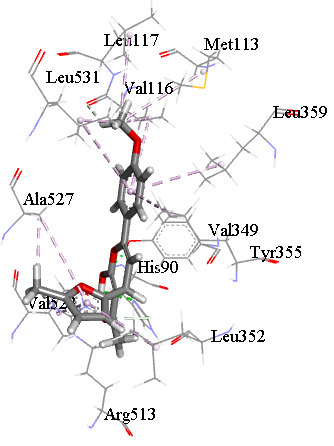 |
| 9a | 9b |


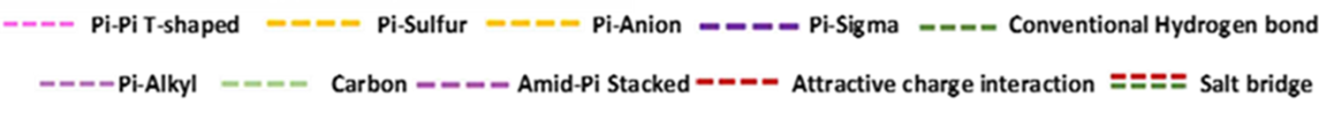

Supplement: Supplementary file 1 — Supplementary Material 1 [file 41598_2025_31238_MOESM1_ESM.docx]
